# AGER1 deficiency-triggered ferroptosis drives fibrosis progression in nonalcoholic steatohepatitis with type 2 diabetes mellitus

**DOI:** 10.1038/s41420-023-01477-z

**Published:** 2023-06-06

**Authors:** Yihui Gong, Zijun Liu, Yuanyuan Zhang, Jun Zhang, Yin Zheng, Zhongming Wu

**Affiliations:** 1 NHC Key Laboratory of Hormones and Development, Chu Hsien-I Memorial Hospital and Tianjin Institute of Endocrinology, Tianjin Medical University, Tianjin, 300134 China; 2 Tianjin Key Laboratory of Metabolic Diseases, Tianjin Medical University, Tianjin, 300134 China; 3 Department of Endocrinology, Shandong Provincial Hospital Affiliated to Shandong First Medical University, Jinan, Shandong 250021 China; 4 Shandong Institute of Endocrine and Metabolic Diseases, Jinan, Shandong 250021 China

**Keywords:** Cell death, Diabetes complications

## Abstract

Hyperglycemia is an independent risk factor for the rapid progression of nonalcoholic steatohepatitis (NASH) to liver fibrosis with an incompletely defined mechanism. Ferroptosis is a novel form of programmed cell death that has been identified as a pathogenic mechanism in various diseases. However, the role of ferroptosis in the development of liver fibrosis in NASH with type 2 diabetes mellitus (T2DM) is unclear. Here, we observed the histopathological features of the progression of NASH to liver fibrosis as well as hepatocyte epithelial-mesenchymal transition (EMT) in a mouse model of NASH with T2DM and high-glucose-cultured steatotic human normal liver (LO2) cells. The distinctive features of ferroptosis, including iron overload, decreased antioxidant capacity, the accumulation of reactive oxygen species, and elevated lipid peroxidation products, were confirmed in vivo and in vitro. Liver fibrosis and hepatocyte EMT were markedly alleviated after treatment with the ferroptosis inhibitor ferrostatin-1. Furthermore, a decrease in the gene and protein levels of AGE receptor 1 (AGER1) was detected in the transition from NASH to liver fibrosis. Overexpression of AGER1 dramatically reversed hepatocyte EMT in high-glucose-cultured steatotic LO2 cells, whereas the knockdown of AGER1 had the opposite effect. The mechanisms underlying the phenotype appear to be associated with the inhibitory effects of AGER1 on ferroptosis, which is dependent on the regulation of sirtuin 4. Finally, in vivo adeno-associated virus-mediated AGER1 overexpression effectively relieved liver fibrosis in a murine model. Collectively, these findings suggest that ferroptosis participates in the pathogenesis of liver fibrosis in NASH with T2DM by promoting hepatocyte EMT. AGER1 could reverse hepatocyte EMT to ameliorate liver fibrosis by inhibiting ferroptosis. The results also suggest that AGER1 may be a potential therapeutic target for the treatment of liver fibrosis in patients with NASH with T2DM.

**Figure:**
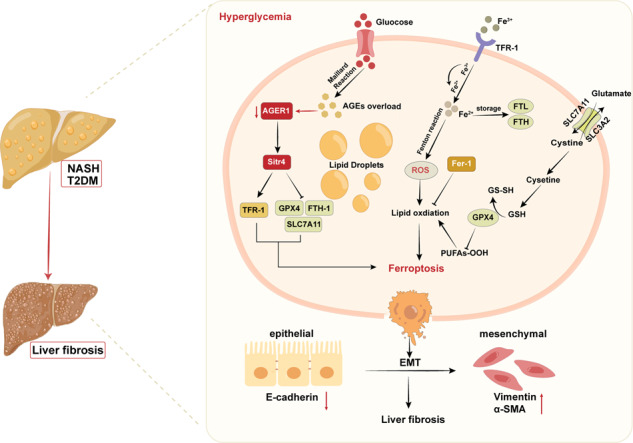
Chronic hyperglycemia is associated with increased advanced glycation end products, resulting in the downregulation of AGER1. AGER1 deficiency downregulates Sirt4, which disturbs key regulators of ferroptosis (TFR-1, FTH, GPX4, and SLC7A11). These lead to increased iron uptake, decreasing the antioxidative capacity and enhanced lipid ROS production, ultimately leading to ferroptosis, which further promotes hepatocyte epithelial-mesenchymal transition and fibrosis progression in NASH with T2DM.

Chronic hyperglycemia is associated with increased advanced glycation end products, resulting in the downregulation of AGER1. AGER1 deficiency downregulates Sirt4, which disturbs key regulators of ferroptosis (TFR-1, FTH, GPX4, and SLC7A11). These lead to increased iron uptake, decreasing the antioxidative capacity and enhanced lipid ROS production, ultimately leading to ferroptosis, which further promotes hepatocyte epithelial-mesenchymal transition and fibrosis progression in NASH with T2DM.

## Introduction

Nonalcoholic fatty liver disease (NAFLD) is the most common chronic disease, affecting 25% of the population worldwide [1], and can progress to nonalcoholic steatohepatitis (NASH), which is characterized by hepatocyte ballooning, cell death, and inflammation [2]. Approximately 41% of NASH patients will further develop liver fibrosis [3], which increases the risk of cirrhosis and hepatocellular carcinoma [4, 5], leading to liver failure and ultimately requiring liver transplantation [6]. Liver fibrosis is the leading determinant of clinical disease prognosis and long-term mortality in NASH patients [7]. Therefore, the prevention and treatment of liver fibrosis are considered a prime focus in NASH.

The pathogenesis of liver fibrosis is the imbalance between synthesis and degradation of the extracellular matrix (ECM), thus resulting in both increased and altered deposition of ECM components. Myofibroblasts are the primary moderators of fibrosis caused by the excess deposition of ECM. Currently, the mechanism underlying the progression of liver fibrosis in NASH is mainly focused on the activation of hepatic stellate cells, which are recognized as the primary sources of myofibroblasts [8]. However, it is now thought that myofibroblasts can be derived from multiple sources, the injured hepatocytes undergoing the epithelial-to-mesenchymal transition (EMT) are also an important source of myofibroblasts in the pathological process of liver fibrosis [9]. Sustained injury or oxidative stress incites the EMT of hepatocytes, in which hepatocytes transform into myofibroblasts, producing the excessive deposition of ECM and ultimately leading to liver fibrosis [10]. EMT is characterized by the loss of epithelial markers (E-cadherin) and the subsequent acquisition of mesenchymal markers (Vimentin, α-SMA, S100A4) [11]. The expression of the mesenchymal marker S100A4 has been reported in the parenchymal cells of patients with NASH-cirrhosis [12]. Hepatocytes derived from mice with liver fibrosis induced by carbon tetrachloride express mesenchymal marker vimentin [13]. In a mouse model of liver fibrosis, 45% of fibroblast-specific protein-1-positive fibroblasts were found to originate from hepatocytes through EMT [14]. Although hepatocyte EMT plays an important role in the pathogenesis of liver fibrosis, the regulatory mechanisms of hepatocyte EMT are complex and not fully understood. In the process of hepatocyte EMT, the protein expression of ferritin heavy chain (FTH) is reduced, and the level of the labile iron pool is increased, accompanied by the accumulation of reactive oxygen species (ROS). Overexpression of FTH or administration of iron chelators or ROS inhibitors protects against hepatocyte EMT [15]. A clinical study found that NAFLD patients with iron overload had a higher fibrosis stage than those without iron overload [16]. Compared with normal mouse livers, fibrotic livers have a significant increase in iron ions and ROS [17]. These aforementioned studies strongly suggest that iron overload and ROS are important factors leading to hepatocyte EMT.

Hyperglycemia has been recognized as an independent risk factor for the accelerated progression of NASH to liver fibrosis [18, 19]; however, the underlying mechanism has not been fully elucidated. Prolonged hyperglycemia in patients with diabetic leads to an increase in reducing sugars and accelerates the formation of advanced glycation end products (AGEs), eventually causing AGEs accumulation [20]. AGEs are destructive heterogeneous molecules that are irreversibly generated during the nonenzymatic glycation process [21]. Due to these oxidative stress or proinflammatory effects, AGEs have been implicated in the pathogenesis of multiple complications of type 2 diabetes mellitus (T2DM) [22]. Recent studies have reported that the cytotoxic effects of accumulated AGEs on hepatocytes result in cell damage, which contributes to the activation of the profibrotic pathway leading to fibrosis progression in NASH mice [23–25]. Increased lipid peroxidation and iron overload, accompanied by a reduction in the ferritin (FTH) level were detected in AGEs-treated mouse cardiomyocytes [26]. Lipid peroxidation, iron overload, and ROS may be important triggers of AGE-induced cell injury; however, the molecular mechanism involved is not well understood. AGE receptor-1 (AGER1) is one of the receptors for AGEs that is responsible for the elimination of AGEs and acts as a negative regulator of oxidative stress and inflammation [27]. In mouse mesangial cells cultured with AGEs, the intracellular ROS level was significantly elevated, which could be reversed by the overexpression of AGER1 [28]. Moreover, the knockdown of AGER1 increased ROS production in a mouse model of premature aging [29]. Therefore, AGER1 dysfunction may be responsible for the cytotoxicity induced by AGEs and appears to play an important role in the molecular mechanisms of liver fibrosis in NASH with T2DM.

In the present work, we performed a range of in vitro and in vivo experiments to investigate the important role of ferroptosis, a novel form of regulated cell death triggered by iron-dependent lipid peroxidation, in the pathogenesis of liver fibrosis in NASH with T2DM, and we further addressed whether AGER1 could improve liver fibrosis in NASH with T2DM by inhibiting ferroptosis. This work provides insights into the mechanism involved in the development of liver fibrosis in NASH with T2DM as well as potential targets for therapeutic strategies.

## Results

### Ferroptosis is involved in liver fibrosis in NASH mice with T2DM

**Fig. 1:**
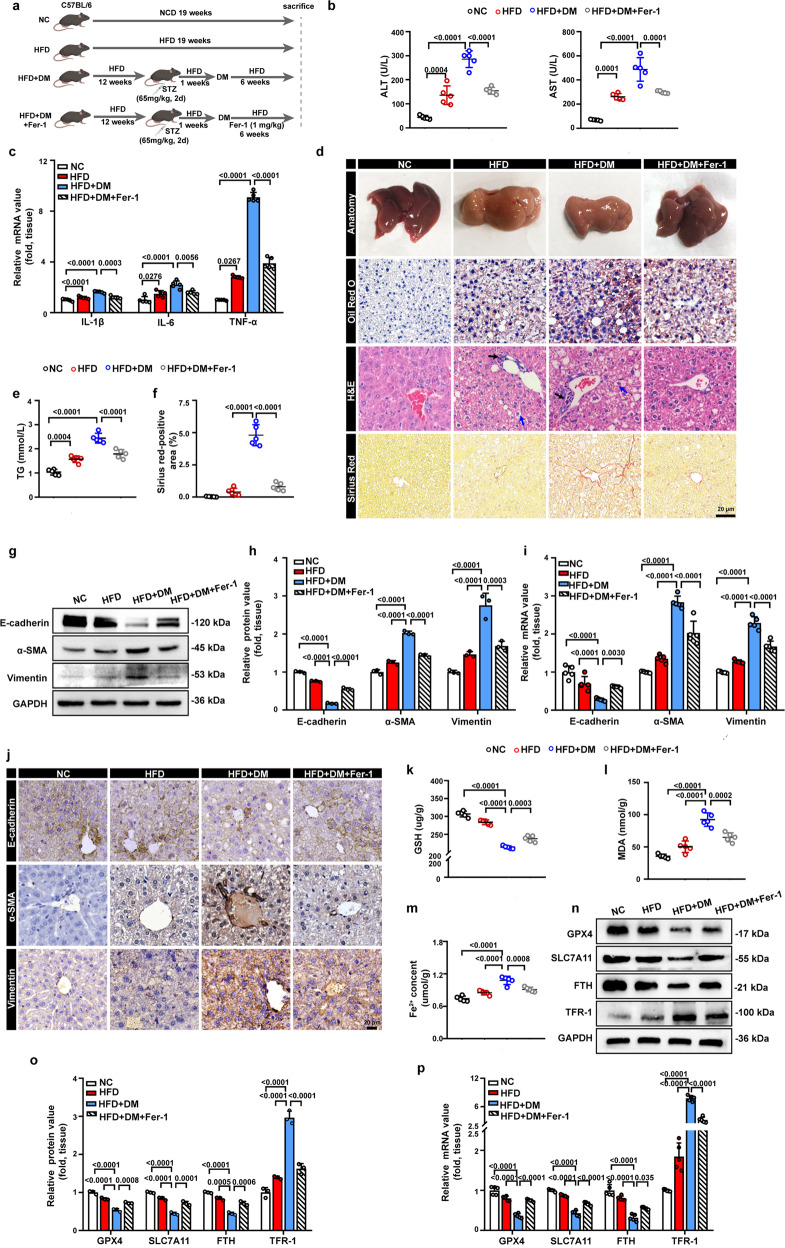
Inhibition of ferroptosis ameliorates liver fibrosis in NASH mice with T2DM. **a** Schematic showing the study design: Male C57BL/6 mice were fed a standard diet of normal chow for 19 weeks (NC group) or were fed a HFD for 19 weeks (HFD). After 12 weeks of high-fat feeding, mice were injected with a low dose of STZ, one week after STZ treatment, the mice with fasting blood glucose levels over 300 mg/dL were characterized as diabetic. And then fed a HFD for another 6 weeks (HFD + DM) or were injected with 1 mg/kg Fer-1 once a day followed by a HFD for 6 weeks (HFD + DM+Fer-1), *n* = 5 mice per group. **b** Serum ALT and AST levels in the indicated groups. **c** The relative mRNA expression of genes associated with the inflammatory cytokines IL-1β, IL-6, and TNF-α in the livers of the indicated groups. **d** Representative pictures of the liver appearance and images of Oil red O staining, H&E, and Sirius Red staining in liver samples from the indicated groups of mice. Black arrows indicate inflammatory cell infiltration, blue arrow indicate hepatocyte ballooning. Scale bars: 20 µm. **e** Serum TG in the indicated groups. **f** Quantification of positive areas of Sirius red measured by ImageJ. **g**, **h** Representative immunoblotting bands and quantitative analysis of the expression alterations of EMT-related indicators, including E-cadherin, α-SMA, and vimentin, in the livers of the indicated groups. **i** The relative mRNA expression of genes associated with EMT-related indicators in the livers of the indicated groups. **j** Representative images of IHC staining for E-cadherin, α-SMA, and vimentin in liver samples from the indicated groups. Scale bars: 20 µm. **k**–**m** Hepatic GSH content, hepatic MDA content, and hepatic Fe^2+^ levels in the indicated groups. **n, o** Representative immunoblotting bands and quantitative analysis of alterations in the expression of ferroptosis indicators, including GPX4, SLC7A11, FTH, and TFR-1, in the livers of the indicated groups. **p** The relative mRNA expression of genes associated with ferroptosis-related indicators in the livers of the indicated groups. Data are expressed as the mean ± SD. All the above experiments were independently repeated at least three times. Statistical significance was tested by one-way ANOVA followed by Tukey’s multiple comparisons post hoc test.

Hyperglycemia has been recognized as an independent risk factor for the development of NASH and is particularly associated with fibrosis [18]. However, the molecular mechanisms involved remain undefined. We established a unique mouse model with a combination of NASH and T2DM (HFD + DM) to explore the mechanism underlying liver fibrosis in NASH with T2DM. The NASH with T2DM mouse model was induced by high-fat diet feeding combined with a low-dose streptozotocin injection. The NASH mouse model (HFD) was generated by feeding mice a high-fat diet alone (Fig. 1a). After 19 weeks of feeding, the HFD + DM and HFD groups exhibited typical characteristics of NASH, i.e., hepatocyte death, pronounced steatosis with ballooning hepatocytes, and liver inflammation. Compared to those of the NC group, the serum levels of alanine aminotransferase (ALT) and aspartate aminotransferase (AST) were increased in the HFD and HFD + DM groups, indicating liver dysfunction and hepatocyte injury (Fig. 1b). The mRNA levels of the inflammation-induced cytokines IL-1β, IL-6, and TNF-α were significantly increased in the livers of the HFD and HFD + DM groups compared with the NC group, suggesting a prominent inflammatory status in the livers of these two models (Fig. 1c). The gross appearance of the livers isolated from the HFD and HFD + DM groups presented a distinct pale white color, which indicated an increased lipid content in the liver tissue (Fig. 1d). Hepatic steatosis was observed in the HFD and HFD + DM groups, as demonstrated by lipid droplets accumulation in liver tissue detected by Oil red O staining (Fig. 1d) as well as increased serum levels of triglyceride (TG) (Fig. 1e). Furthermore, the results of H&E staining displayed remarkable hepatocyte ballooning in the HFD and HFD + DM groups (Fig. 1d). Sirius red staining was used for the assessment of liver fibrosis. We found that the mouse model of NASH did not develop liver fibrosis, as almost no collagen accumulation was detected by Sirius red staining, consistent with previous research, which also demonstrated that a long-term HFD could cause mild pathological features of NASH but rarely fibrosis [30]. However, excessive collagen fiber deposition was observed in the NASH with T2DM mouse model, indicating that liver fibrosis was induced in this model (Fig. 1d, f). In view of the critical role of hepatocyte EMT in the pathogenesis of liver fibrosis [31, 32], we further investigated whether hepatocyte EMT was involved in liver fibrosis in NASH with T2DM. The expression of EMT-related indicators was detected in hepatic tissue, as confirmed by WB and qPCR analysis. The expression levels of the epithelial marker E-cadherin were significantly decreased, and the expression levels of the mesenchymal markers α-smooth muscle actin (α-SMA) and vimentin were markedly increased in the HFD + DM group compared with the HFD and NC groups, indicating that hepatocyte lost the epithelial features and acquired a mesenchymal phenotype (Fig. 1g–i). The immunohistochemistry results displayed a similar trend to those of the WB and qPCR analyses (Fig. 1j). All of these findings suggested that hepatocyte EMT and liver fibrosis were induced in the mouse model of NASH with T2DM.

Accumulation of ROS and iron overload have been verified to induce the EMT of hepatocytes [33, 34]. Therefore, we hypothesized that ferroptosis, a novel form of programmed cell death characterized by the accumulation of ROS in an iron-dependent manner [35], might contribute to hepatocyte EMT and liver fibrosis. In the present work, significantly reduced antioxidant enzyme levels of glutathione (GSH) were detected in the HFD + DM group compared with the HFD and NC groups, indicating an inactivated antioxidant system in HFD + DM mice (Fig. 1k). The lipid peroxidation product level was also significantly elevated in the HFD + DM group, as indicated by malondialdehyde (MDA) analysis (Fig. 1l). Moreover, the Fe^2+^ content was increased in the liver of the HFD + DM group, suggesting iron overload in liver tissues (Fig. 1m). These findings indicated that the liver tissues of the HFD + DM group presented a lower antioxidant capacity, higher lipid peroxidation, and iron overload, which are hallmarks of ferroptosis. The expression of key regulators of ferroptosis-related pathways, including glutathione peroxidase 4 (GPX4) and solute carrier family 7 member 11 (SLC7A11), which are important components of the antioxidant system [36], and two iron metabolism-related molecules responsible for iron storage [37] FTH and iron uptake [38] transferrin receptor 1 (TFR-1), were assessed. As shown in Fig. 1n–p, the protein and mRNA levels of GPX4, SLC7A11, and FTH were dramatically reduced, and the expression levels of TFR-1 were increased in the HFD + DM group relative to the HFD and NC groups. Furthermore, the ferroptosis-related changes in HFD + DM mice were reversed upon treatment with the ferroptosis antagonist ferrostatin-1 (Fer-1) (Fig. 1k–p). The serum ALT and AST levels (Fig. 1b) and mRNA levels of inflammation-induced cytokines were markedly decreased after Fer-1 treatment (Fig. 1c), which further demonstrated the improvement in liver function and hepatic inflammation. Moreover, Fer-1 treatment markedly reduced the Sirius red-stained collagen area, indicating attenuation of liver fibrosis (Fig. 1d, f). Simultaneously, the EMT of hepatocytes was also reversed after the administration of Fer-1, as demonstrated by the expression levels of EMT-related markers (Fig. 1g–j). The above data demonstrated that ferroptosis contributed to the progression of NASH to fibrosis by promoting hepatocyte EMT.

### Ferroptosis and EMT are induced by high glucose in steatotic LO2 cells

**Fig. 2:**
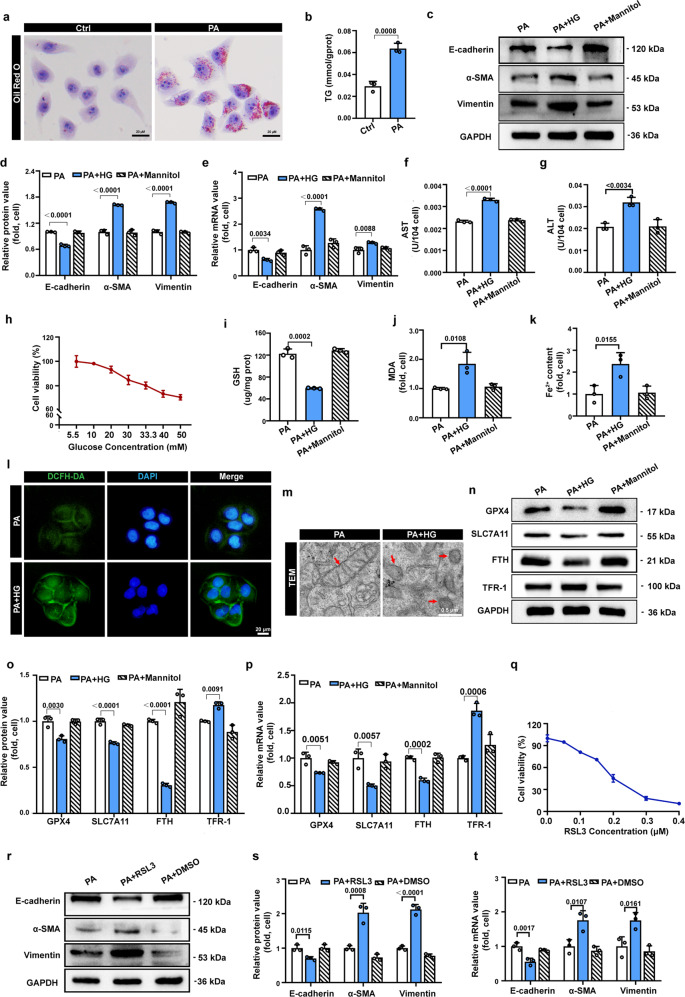
Ferroptosis and EMT are triggered by high glucose in steatotic LO2 cells. **a** Representative images of Oil red O staining of LO2 cells before and after incubation with 110 μM PA. Scale bars: 20 µm. **b** Intracellular triglyceride content in Ctrl cells and PA cells. **c, d** Representative immunoblotting bands and quantitative analysis of expression alterations of EMT-related indicators in the indicated groups of cells. **e** The relative mRNA expression of genes associated with EMT-related indicators in the indicated groups of cells. **f, g** Cellular supernatant AST and ALT contents in the indicated groups. **h** The cell viability of steatotic LO2 cells cultured with various concentrations of glucose for 24 h. **i–k** Intracellular GSH, MDA, and Fe^2+^ levels in the indicated groups. **l** Representative fluorescence images of the indicated group of cells stained with DCFH-DA to detect intracellular ROS (green) and DAPI (blue). **m** Representative images of transmission electron microscopy (TEM)-indicated morphological changes in the mitochondria in the indicated groups of cells. Red arrows indicate mitochondria. Scale bars: 0.5 µm. **n**, **o** Representative immunoblotting bands and quantitative analysis of alterations in the expression of ferroptosis indicators in the indicated groups of cells. **p** The relative mRNA expression of genes associated with ferroptosis-related indicators in the indicated groups of cells. **q** The cell viability of steatotic LO2 cells cultured with various concentrations of RSL3 for 24 h. **r**, **s** Representative immunoblotting bands and quantitative analysis of alterations in the expression of EMT-related indicators in the indicated groups of cells. **t** The relative mRNA expression of genes associated with EMT-related indicators in the indicated groups of cells. Data are expressed as the mean ± SD. All the above experiments were independently repeated at least three times. Statistical significance was tested by Student’s *t* test or one-way ANOVA followed by Tukey’s multiple comparisons post hoc test.

Based on the in vivo validation experiments, we further explored whether EMT and ferroptosis could be induced in high-glucose-cultured steatotic human normal liver (LO2) cells. Considering that lipids are a major contributing factor to steatohepatitis [39], we used palmitic acid (PA) to induce the steatohepatitis cell model. According to Oil red O staining and TG content analysis, numerous lipid droplets and lipid accumulation were observed in LO2 cells following 24 h treatment with PA, indicating the successful establishment of the steatohepatitis cell model (Fig. 2a, b). Next, we explored whether EMT was induced by high glucose in steatotic LO2 cells. As shown in Fig. 2c–e, the protein and mRNA expression levels of the epithelial marker E-cadherin were reduced, and the expression of the mesenchymal markers α-SMA and vimentin was increased in PA + HG cells compared with PA cells. Moreover, there were 1.5 times higher levels of AST and ALT in PA + HG cells than in PA cells, indicating hepatocyte damage in high-glucose-cultured steatotic LO2 cells (Fig. 2f, g). All these results indicated that hepatocyte EMT was induced by high glucose in steatotic LO2 cells, which is consistent with the findings of the in vivo experiments.

We further investigated whether ferroptosis was induced in high-glucose-cultured steatotic LO2 cells. The CCK8 assay results showed that the cell viability dropped below 80% when the concentration of glucose was increased above 33.3 mM, suggesting that high-glucose induced steatotic LO2 cell death (Fig. 2h). As shown in Fig. 2i–k, a dramatically reduced level of GSH, an elevated level of lipid peroxidation (MDA), and increased Fe^2+^ content were detected in the PA + HG cells, which indicated that ferroptosis was triggered by high glucose in steatotic LO2 cells. DCFH-DA staining revealed that PA + HG cells exhibited dramatically increased levels of cytosolic ROS, which are essential factors for the execution of ferroptosis (Fig. 2l). Furthermore, as detected by transmission electron microscopy, typical mitochondrial morphological hallmarks of ferroptosis were observed in PA + HG cells, including the diminution or disappearance of mitochondrial cristae along with increased mitochondrial membrane densities and volume reduction (Fig. 2m). The expression levels of ferroptosis-related indicators were assessed by immunoblot and qPCR analysis. As shown in Fig. 2n–p, lower protein and mRNA expression levels of GPX4, SLC7A11, and FTH and higher expression levels of TFR-1 were detected in PA + HG cells than in PA cells, indicating compromised antioxidant capacity and dysregulated iron metabolism in high-glucose-cultured steatotic LO2 cells. Mannitol was used as an osmotic control of high glucose, and no significant effects were observed in the above parameters in the osmotic control group, which could rule out the effect of high osmolarity (Fig. 2d–g, i–k, n–p). To further verify that high-glucose‐induced steatotic LO2 cell EMT was due to ferroptosis, we treated steatotic LO2 cells with the ferroptosis inducer RSL3. The results of the CCK8 assay revealed that the cell viability dropped below 80% when the concentration of RSL3 was higher than 0.1 μM, indicating that cell death was induced by RSL3 (Fig. 2q). We further explored whether EMT was induced by the ferroptosis inducer RSL3 in steatotic LO2 cells. As shown in Fig. 2r–t, the protein and mRNA expression levels of the epithelial marker E-cadherin were reduced, and the expression levels of the mesenchymal markers α-SMA and vimentin was increased in PA + RSL3 cells compared with PA cells, indicating that hepatocyte EMT was induced by RSL3 in steatotic LO2 cells. Taken together, these data suggest that EMT and ferroptosis were induced in high-glucose-cultured steatotic LO2 cells, and that ferroptosis was involved in the pathological process of hepatocyte EMT.

### Inhibition of ferroptosis in high glucose cultured steatotic LO2 cells

**Fig. 3:**
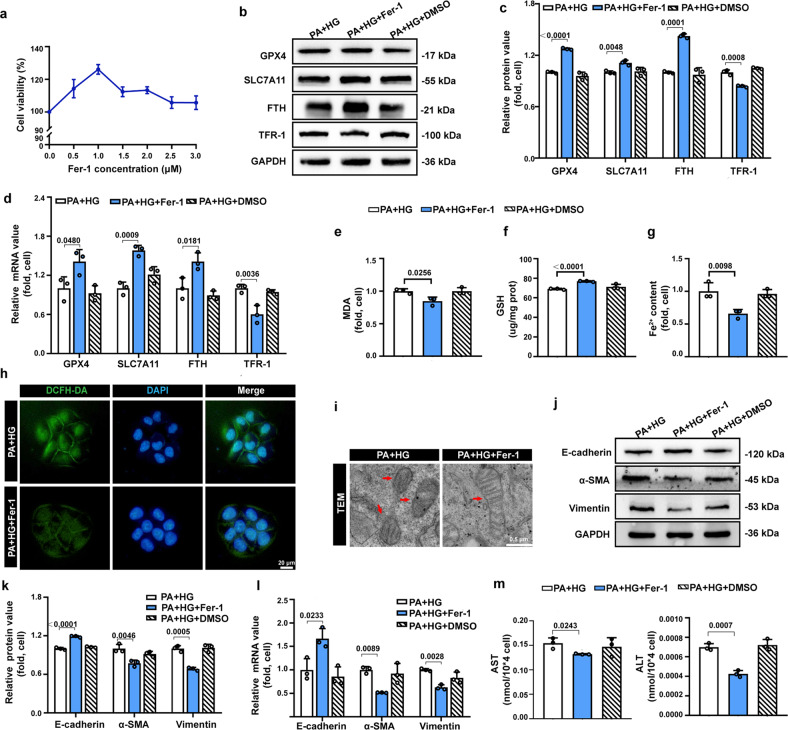
Inhibition of ferroptosis reverses high-glucose-induced EMT in steatotic LO2 cells. **a** The cell viability of steatotic LO2 cells cultured in 33.3 mmol/L glucose medium with different concentrations of Fer-1 for 24 h. **b, c** Representative immunoblotting bands and quantitative analysis of alterations in the expression of ferroptosis-related indicators in the indicated groups of cells. **d** The relative mRNA expression of genes associated with ferroptosis-related indicators in the indicated groups of cells. **e**, **f**, **g** Intracellular MDA, GSH, and Fe^2+^ levels in the indicated groups. **h** Representative fluorescence images of the indicated group of cells stained with DCFH-DA to detect intracellular ROS (green) and DAPI (blue). **i** Representative images of transmission electron microscopy (TEM)-indicated morphological changes in the mitochondria in the indicated groups of cells. Red arrows indicate mitochondria. Scale bars: 0.5 µm. **j**, **k** Representative immunoblotting bands and quantitative analysis of expression alterations of EMT indicators in the indicated groups of cells. **l** The relative mRNA expression of genes associated with EMT indicators in the indicated groups of cells. **m** Cellular supernatant AST and ALT in the indicated groups. Data are expressed as the mean ± SD. All the above experiments were independently repeated at least three times. Statistical significance was tested by one-way ANOVA followed by Tukey’s multiple comparisons post hoc test.

As confirmed in vivo, inhibiting ferroptosis could alleviate liver fibrosis by reversing the EMT of hepatocytes. We further investigated whether ferroptosis inhibitor Fer-1 could mitigate high-glucose-induced hepatocyte EMT in steatotic LO2 cells. After treatment with the different concentrations of Fer-1, the cell viability was increased in all Fer-1 treated groups and reached a peak when the concentration of Fer-1 reached 1 μM, which also indicated that Fer-1 can reduce high glucose-induced cell death (Fig. 3a). The ferroptosis-related changes induced by high glucose in steatotic LO2 cells were reversed after treatment with ferroptosis inhibitor Fer-1 (Fig. 3b–i). Notably, high-glucose-induced hepatocyte EMT was mitigated by the administration of Fer-1, as demonstrated by western blotting and qPCR. The expression of the epithelial marker E-cadherin was elevated, and the expression of mesenchymal markers α-SMA and vimentin was decreased (Fig. 3j–l). AST and ALT levels were also reduced following Fer-1 treatment, indicating that high-glucose-induced hepatocyte damage was also alleviated (Fig. 3m). DMSO served as the solvent control, and we found that DMSO had no significant effects on the above parameters, which excluded the interference of solvent (Fig. 3b–g, j–m). These findings revealed that inhibiting ferroptosis hindered high-glucose-induced hepatocyte EMT in steatotic LO2 cells, in accordance with the in vivo results.

### AGER1 deficiency potentiates ferroptosis sensitivity and exacerbates high-glucose-induced hepatocyte EMT

**Fig. 4:**
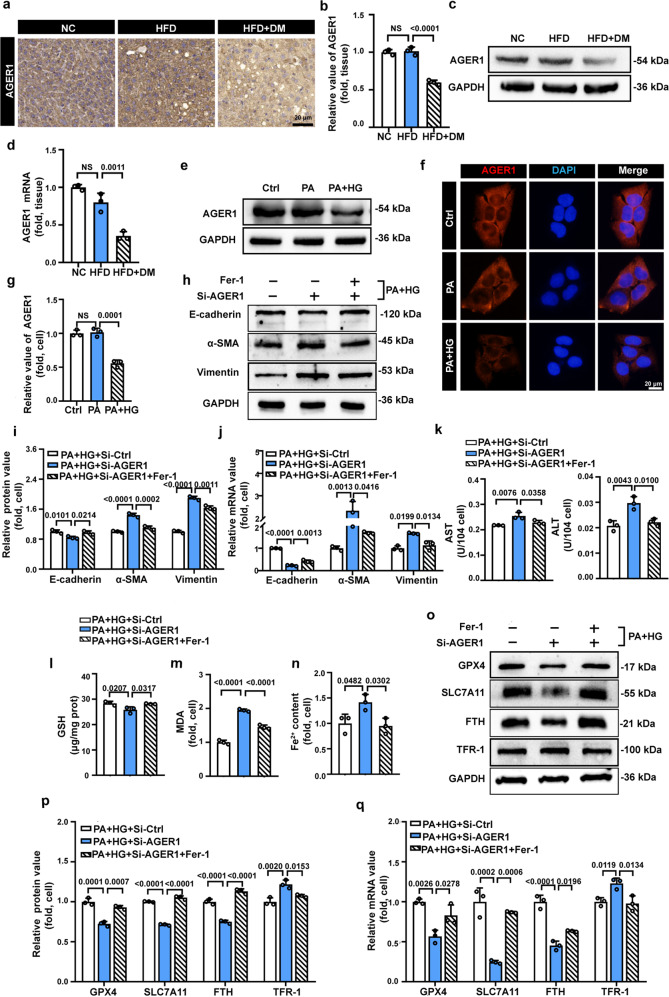
AGER1 knockdown exacerbates high-glucose-induced EMT in steatotic LO2 cells by accelerating ferroptosis. **a** Representative images of IHC staining for AGER1 in liver samples from NC, HFD, and HFD + DM mice. Scale bars: 20 µm. **b, c** Representative immunoblotting bands and quantitative analysis of AGER1 expression alterations in NC, HFD, and HFD + DM mice. **d** The relative mRNA expression of AGER1 in NC, HFD, and HFD + DM mice. **e** Representative immunoblotting bands of AGER1 expression alterations in Ctrl, PA, and PA + HG cells. **f** Representative immunofluorescence images of AGER1 in the indicated groups of cells, AGER1 (red), DAPI (blue). **g** Quantitative analysis of AGER1 expression alterations in Ctrl, PA, and PA + HG cells. **h, i** Representative immunoblotting bands and quantitative analysis of expression alterations of EMT-related indicators in the indicated groups of cells. **j** The relative mRNA expression of EMT-related indicators in the indicated groups of cells. **k** Cellular supernatant ALT and AST in the indicated groups. **l–n** Intracellular GSH, MDA, and Fe^2+^ levels in the indicated groups. **o**, **p** Representative immunoblotting bands and quantitative analysis of alterations in the expression of ferroptosis-related indicators in the indicated groups of cells. **q** The relative mRNA expression of ferroptosis-related indicators in the indicated groups of cells. Data are expressed as the mean ± SD. All the above experiments were independently repeated at least three times. Statistical significance was tested by one-way ANOVA followed by Tukey’s multiple comparisons post hoc test.

Prolonged hyperglycemia in diabetes contributes to the accumulation of AGEs [40]. AGEs accumulation is considered to have profibrogenic activity in NASH [25]. AGER1 is one of the receptors for AGEs that binds, degrades, and eliminates AGEs [27]. A recent clinical study found that the clearance receptor AGER1 was reduced in patients with NASH + DM but not in those with steatosis [41]. However, whether AGER1 is associated with liver fibrosis in NASH and T2DM remains unclear. We first examined the expression levels of AGER1 in the liver tissues of the HFD + DM, HFD, and NC groups. As demonstrated by immunohistochemistry and western blotting, there was similar protein expression of AGER1 in the HFD and NC groups but markedly decreased expression in the HFD + DM group, which was also confirmed by qPCR analysis (Fig. 4a–d). Consistent with the results of the in vivo experiments, AGER1 expression was markedly decreased in PA + HG cells, whereas there was no difference between NC and PA cells (Fig. 4e–g).

These results suggested that AGER1 deficiency might be a predisposing factor for the progression of NASH to liver fibrosis. Therefore, we conducted AGER1 loss-of-function experiments to explore the effect of AGER1 on hepatocyte EMT. The successful knockdown of AGER1 was verified by western blotting and qPCR analysis (Supplementary Fig. 1a–c). According to the changes in the protein and mRNA expression levels of EMT-related indicators (E-cadherin, α-SMA, and vimentin), high-glucose-induced hepatocyte EMT in steatotic LO2 cells was strikingly aggravated by AGER1 knockdown (Fig. 4h–j). Moreover, the hepatocyte damage-related indicators AST and ALT were markedly increased by AGER1 knockdown (Fig. 4k). These data confirm that AGER1 plays a protective role in hepatocyte EMT and that AGER1 deficiency exacerbates high-glucose-induced hepatocyte EMT in steatotic LO2 cells.

Based on the validation from in vivo and in vitro experiments that ferroptosis plays a significant role in the initiation of hepatocyte EMT, we further investigated the effect of AGER1 on ferroptosis. As shown in Fig. 4l–n, high-glucose-induced decreased antioxidant defenses, the accumulation of lipid peroxidation, and iron overload were all aggravated by the knockdown of AGER1. Western blotting and qPCR analysis confirmed that the expression profiles of ferroptosis-related indicators, including GPX4, SLC7A11, and FTH, were notably decreased and the expression of TFR-1 was observably increased by AGER1 knockdown (Fig. 4o–q). In addition, the ferroptosis antagonist Fer-1 reversed AGER1 deficiency-induced ferroptosis changes (Fig. 4l–q). These results demonstrate that ferroptosis was triggered by AGER1 knockdown. More notably, the AGER1 deficiency-induced exacerbation of hepatocyte EMT and hepatocyte damage was improved after Fer-1 treatment (Fig. 4h–k). Thus, the above data indicated that the exacerbated hepatocyte EMT caused by AGER1 deficiency may be due to its promotion of ferroptosis.

### AGER1-mediated suppression of ferroptosis is Sirt4 dependent

**Fig. 5:**
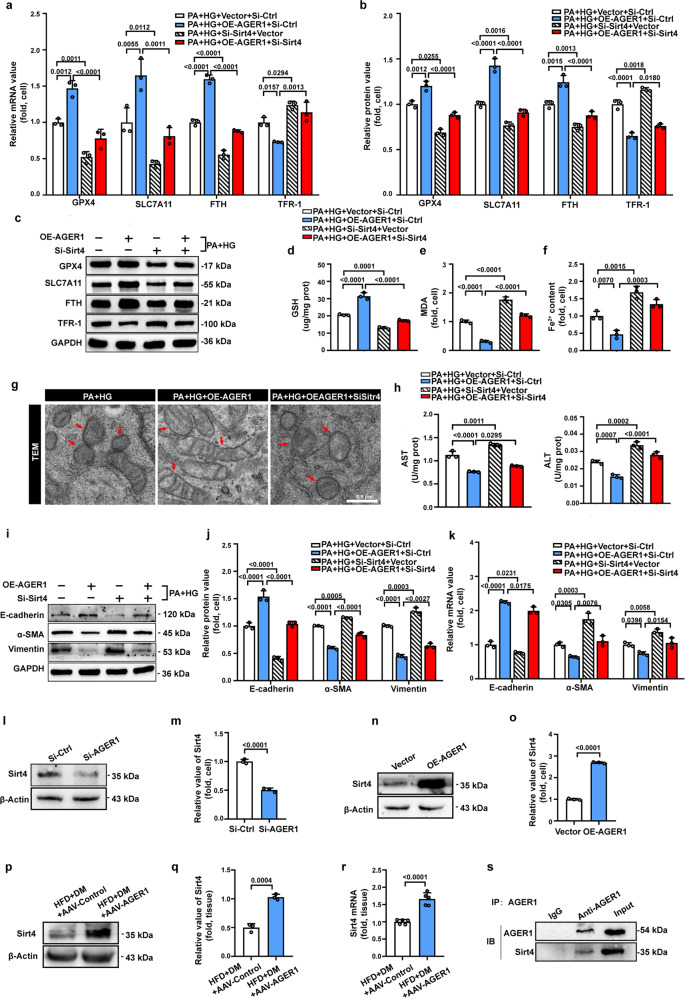
AGER1-mediated suppression of ferroptosis is Sirt4 dependent. **a** The relative mRNA expression of ferroptosis-related indicators in the indicated groups of cells. **b**, **c** Representative immunoblotting bands and quantitative analysis of alterations in the expression of ferroptosis-related indicators in the indicated groups of cells. **d–f** Intracellular GSH, MDA, and Fe^2+^ levels in the indicated groups. **g** Representative images of transmission electron microscopy (TEM)-indicated morphological changes in the mitochondria in the indicated groups of cells. Red arrows indicate mitochondria. Scale bars: 0.5 µm. **h** Cellular supernatant AST and ALT in the indicated groups. **i**, **j** Representative immunoblotting bands and quantitative analysis of expression alterations of EMT-related indicators in the indicated groups of cells. **k** The relative mRNA expression of EMT-related indicators in the indicated groups of cells. **l, m** Representative immunoblotting bands and quantitative analysis of expression alterations of Sirt4 in Si-Ctrl and Si-AGER1 cells. **n**, **o** Representative immunoblotting bands and quantitative analysis of expression alterations of Sirt4 in Vector and OE-AGER1 cells. **p**, **q** Representative immunoblotting bands and quantitative analysis of expression alterations of Sirt4 in HFD + DM + AAV-Control and HFD + DM + AAV-AGER1 mice. **r** The relative mRNA expression of Sirt4 in HFD + DM + AAV-Control and HFD + DM + AAV-AGER1 mice. **s** Representative immunoblotting bands for coimmunoprecipitation (co-IP) detection in LO2 cells incubated with PA and high glucose. IP immunoprecipitation, IB immunoblotting. Data are expressed as the mean ± SD. All the above experiments were independently repeated at least three times. Statistical significance was tested by Student’s *t* test or one-way ANOVA followed by Tukey’s multiple comparisons post hoc test.

AGER1 loss-of-function experiments confirmed the modulatory effect of AGER1 on ferroptosis and hepatocyte EMT. We further performed gain-of-function experiments to validate this effect. The overexpression efficiency of AGER1 was confirmed by western blotting and qRT‒PCR analysis (Supplementary Fig. 1d–f). The functional gain of AGER1 dramatically rescued the ferroptosis-related changes induced by high glucose in steatotic LO2 cells (Fig. 5a–g). Simultaneously, hepatocyte injury and hepatocyte EMT were also alleviated by AGER1 overexpression (Fig. 5h–k). These results further illustrate that AGER1 acts as a suppressor of ferroptosis.

Given the pronounced prohibitive function of AGER1 on ferroptosis, we next sought to understand how AGER1 signaling suppresses ferroptosis. We reasoned that sirtuin 4 (Sirt4) may be a key signaling intermediate in this process. Sirt4 is an NAD^+^-dependent deacetylase that is highly expressed in the liver [42]. Previous studies indicated that Sirt4 could reduce ROS production [43], the key driver of ferroptosis. Furthermore, Sirt4 has been shown to repress glutamine metabolism, which is required for ferroptosis [44]. As an initial test of this hypothesis, we investigated the influence of AGER1 on Sirt4. As demonstrated by western blotting, the protein level of Sirt4 was markedly decreased by the knockdown of AGER1 and increased by AGER1 overexpression in LO2 cells (Fig. 5l–o). Similarly, the protein and mRNA levels were also significantly increased in the AGER1 overexpression mouse model group (AAV-AGER1) compared with the AAV-Control group (Fig. 5p–r). Furthermore, co-IP experiments revealed that Sirt4 physically interacted with AGER1 (Fig. 5s). The above results show that Sirt4 might be a target gene of AGER1. Thereafter, we examined whether the knockdown of Sirt4 produces a ferroptosis phenotype analogous to that of AGER1 knockdown. Successful knockdown of Sirt4 was detected by qPCR analysis (Supplementary Fig. 1g). Western blotting and qPCR analysis indicated that the expression of ferroptosis-inhibiting factors (GPX4, SLC7A11, and FTH) was notably decreased, and the expression of the ferroptosis-promoting factor TFR-1 was markedly increased by Sirt4 knockdown (Fig. 5a–c). The decreased antioxidant capacity, elevated lipid peroxidation products, and iron overload detected in high-glucose-cultured steatotic LO2 cells were significantly accelerated by Sirt4 dysfunction (Fig. 5d–f). The results showed that Sirt4 plays a similar role as AGER1 in the inhibition of ferroptosis.

Based on the above results, we hypothesized that the inhibitory effect of AGER1 on ferroptosis may be dependent on the regulation of Sirt4. To confirm this conjecture, we performed a cotransfection experiment. As shown, the ameliorating effect of AGER1 overexpression on ferroptosis was abrogated upon Sirt4 ablation (Fig. 5a–f). The hallmarks of ferroptosis, including the diminution or disappearance of mitochondrial cristae, increased mitochondrial membrane densities and volume reduction, were mitigated by AGER1 overexpression but reversed by cotransfection with Sirt4 siRNA (Fig. 5g). In addition, the elimination effect of AGER overexpression on hepatocyte injury and hepatocyte EMT was also significantly weakened upon Sirt4 ablation (Fig. 5h–k). Taken together, these results demonstrate that Sirt4 is involved in the inhibitory effect of AGER1 on ferroptosis.

### AGER1 overexpression alleviates liver fibrosis in NASH mice with T2DM

**Fig. 6:**
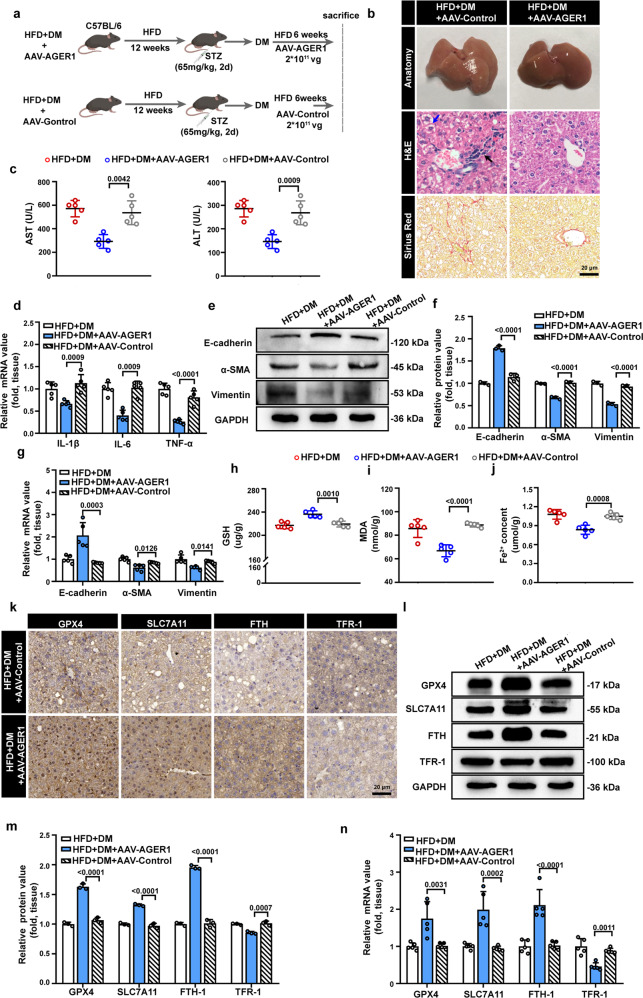
Overexpression of AGER1 ameliorates liver fibrosis in NASH mice with T2DM. **a** Schematic showing the study design: Male C57BL/6 mice were fed a HFD for 12 weeks and were then injected with a low dose of STZ to induce diabetes. After the NASH + T2DM mouse model was established, mice were injected with AAV9-AGER1 (HFD + DM + AAV-AGER1) or AAV-GFP (HFD + DM + AAV-Control) and then were fed a HFD for an additional 6 weeks, n = 5 mice per group. **b** Representative pictures of the liver appearance and images of H&E and Sirius red staining in liver samples from the HFD + DM + AAV-Control group and HFD + DM + AAV-AGER1 group. Black arrows indicate inflammatory cell infiltration, blue arrow indicate hepatocyte ballooning. Scale bars: 20 µm. **c** Serum AST and ALT levels. **d** The relative mRNA expression of genes associated with the inflammatory cytokines IL-1β, IL-6 and TNF-α in the livers of the indicated groups. **e**, **f** Representative immunoblotting bands and quantitative analysis of expression alterations of EMT indicators in the livers of the indicated groups of mice. **g** The relative mRNA expression of genes associated with EMT-related indicators in the livers of the indicated groups. **h–j** Hepatic GSH, MDA, and Fe^2+^ levels in the indicated groups. **k** Representative images of IHC staining for GPX4, SLC7A11, FTH, and TFR-1 in liver samples from the HFD + DM + AAV-Control and HFD + DM + AAV-AGER1 groups. Scale bars: 20 µm. **l**, **m** Representative immunoblotting bands and quantitative analysis of alterations in the expression of ferroptosis indicators in the livers of the indicated groups. **n** The relative mRNA expression of genes associated with ferroptosis-related indicators in the livers of the indicated groups. Data are expressed as the mean ± SD. All the above experiments were independently repeated at least three times. Statistical significance was tested by one-way ANOVA followed by Tukey’s multiple comparisons post hoc test.

Based on the inhibitory effect of AGER1 on hepatocyte EMT, as confirmed by in vitro experiments, we further investigated the influence of AGER1 on liver fibrosis in vivo. Following the establishment of the NASH with T2DM mouse model, mice were subjected to tail vein infection with AAV9-AGER1 or AAV9-GFP (Control) followed by HFD feeding for another 6 weeks (Fig. 6a). Successful overexpression of AGER1 was detected by western blotting and qPCR analysis (Supplementary Fig. 1h-j). Compared to those in the AAV-Control group, liver steatosis and fibrosis were mitigated in the AAV-AGER1 group, as revealed by H&E staining and Sirius red staining (Fig. 6b). In serum biochemical analyses, serum AST and ALT levels were markedly reduced in the AAV-ACER1 group, which further indicated the improvement in liver function (Fig. 6c). The mRNA levels of the inflammation-induced cytokines IL-1β, IL-6, and TNF-α were significantly decreased in the livers of the AAV-AGER1 group, suggesting that hepatic inflammation was mitigated by AGER1 overexpression (Fig. 6d). Moreover, we detected EMT-related indicators in liver tissues. As shown in Fig. 6e–g, hepatocyte EMT was reversed by AGER1 overexpression. Consistent with the in vitro experiments, mice with AGER1 overexpression showed increased GSH levels and decreased intrahepatic Fe^2+^ and MDA levels (Fig. 6h–g). We further assessed the expression levels of ferroptosis-related markers in mouse livers. The the expression of GPX4, SLC7A11, and FTH was enhanced, and TFR-1 was decreased in the AAV-AGER1 group, as confirmed by IHC staining, western blotting and qPCR analysis (Fig. 6k–n). The above results suggest that AGER1 alleviates liver fibrosis and hepatocyte EMT by regulating ferroptosis in NASH with T2DM.

## Discussion

Clinically, hyperglycemia appears to be the most significant clinical predictor for the rapid progression of NASH to adverse hepatic outcomes, particularly liver fibrosis [45]. Liver fibrosis is a major area of focus in NASH research since it is the main determinant of mortality in patients with NASH and has no approved therapeutic agents [46]. Therefore, a detailed understanding of the pathogenic mechanism underlying the progression of liver fibrosis in NASH driven by T2DM and identifying potential signaling regulators to prevent NASH progression have become imperative issues. Herein, we established a mouse model of NASH with T2DM and demonstrated that ferroptosis is involved in the pathogenesis of liver fibrosis by promoting hepatocyte EMT. We identified AGER1 as a protective regulator against liver fibrosis, and overexpression of AGER1 alleviated hepatocyte EMT and liver fibrosis. The mechanisms underlying the phenotypes appear to be associated with the inhibitory influence of AGER1 on ferroptosis, which is dependent on the regulation of Sirt4, and suggest that AGER1 is a useful therapeutic target to prevent fibrosis progression in patients with NASH with T2DM.

The methionine-and choline-deficient (MCD) diet is commonly used to induce a rapid onset of the NASH phenotype [47]. However, the MCD diet leads to weight loss and fails to cause insulin resistance, thus failing to fully mimic the symptoms of NASH patients [48]. In addition, some genetic models, such as ob/ob mice or db/db mice, exhibit typical pathologies of the NASH phenotype such as obesity and insulin resistance. However, hyperglycemia and NASH occur simultaneously, which makes it impossible to assess the effects of diabetes on preexisting NASH [49, 50]. In the present work, we established a mouse model of NASH with T2DM by HFD combined with a low-dose STZ injection, which was more representative of human disease and could be used to investigate the effect of diabetes on preexisting NASH.

Persistent hyperglycemia in diabetes can initiate actions that lead to organ dysfunction and multiple complications, such as diabetic nephropathy and cardiovascular disease [51], and recent studies have shown that ferroptosis is involved in the development of these complications [52–54]. Ferroptosis-mediated cell death leads to dying HK2 cells (human proximal tubular cell line) secreting profibrotic factors and facilitating fibroblast proliferation and activation, which promotes renal fibrosis in diabetic nephropathy [55]. The role of ferroptosis in the progression of liver fibrosis exacerbated by diabetes remains elusive. Nelson et al. reported that hepatic iron deposition is associated with advanced histologic features such as fibrosis in NASH [56]. Consistently, NASH patients with a mutation in the HFE gene, which can lead to iron overload, have more severe hepatic fibrosis than those without this mutation [57]. Another group confirmed that pioglitazone can improve liver fibrosis in NASH patients, which is related to the inhibition of the ferroptosis pathway [58]. Here, we demonstrated that ferroptosis was triggered during fibrosis progression in a NASH with T2DM mouse model as well as in high-glucose-cultured steatotic LO2 cells. Furthermore, additional treatment with Fer-1, an inhibitor of ferroptosis, markedly attenuated liver fibrosis and hepatocyte EMT in vivo and in vitro. Based on the results from our analysis and previous reports, ferroptosis is involved in the mechanisms of liver fibrosis induced by diabetes in NASH.

EMT is a dynamic transition in which epithelial cells exhibit altered cellular architecture and lose adherens junctions, eventually transforming into mesenchymal cells [59]. Research has demonstrated that hepatocyte EMT represents an important pathogenic pathway in liver fibrosis [60, 61]. In the present work, we found that hepatocyte EMT participates in the pathological process of liver fibrosis in this model. Furthermore, hepatocyte mesenchymal morphology in vivo and in vitro was markedly attenuated by treatment with the ferroptosis inhibitor Fer-1. Previous studies have confirmed that the accumulation of ROS can initiate the EMT program through reversible or irreversible oxidative modifications of the key protein involved in the EMT process [62]. Mehta et al. reported that the induction of EMT was dependent on iron metabolism, and excess iron exposure led to elevated expression of the mesenchymal markers vimentin and N-cadherin in HepG2 cells [34]. Taken together, these data indicate that ferroptosis is involved in the pathogenesis of liver fibrosis by regulating hepatocyte EMT.

Chronic hyperglycemia in diabetes can promote the formation and accumulation of AGEs [63, 64]. As the receptor of AGEs, AGER1 is responsible for the elimination of AGEs, but under sustained excess of AGEs, the expression and function of AGER1 are disordered [27]. AGER1 could be a therapeutic target in diabetes-related vascular and kidney disease due to its anti-inflammatory and antioxidative stress effects [28, 65]. However, the physiological function of AGER1 has largely remained unknown. In the present work, we found that the protein and gene expression levels of AGER1 were robustly decreased in the livers of NASH mice with T2DM and in high-glucose-cultured steatotic LO2 cells. Moreover, knockdown of AGER1 strikingly aggravates hepatocyte EMT, while overexpression of AGER1 markedly alleviates hepatocyte EMT and liver fibrosis. All of these results indicate that the downregulation of AGER1 is responsible for the progression of liver fibrosis in NASH with T2DM. Consistently, knockdown of AGER1 in tubular epithelial cells can induce the process of EMT and promote renal fibrosis [66]. Another study showed that curcumin can effectively alleviate hepatic fibrosis by regulating AGER1 expression [67]. An interesting finding is that the aggravated hepatocyte EMT induced by AGER1 knockdown was reversed by the ferroptosis inhibitor Fer-1, indicating that ferroptosis was involved in the mechanisms of AGER1 knockdown-induced aggravated hepatocyte EMT. Moreover, the hallmarks of ferroptosis were suppressed or exacerbated following AGER1 overexpression or knockdown, which further verified the regulatory effect of AGER1 on ferroptosis. In conclusion, our study has broadened the understanding of AGER1, confirming the protective role of AGER1 in the progression of liver fibrosis, and the mechanism behind the phenotype is related to the inhibitory influence of AGER1 on ferroptosis.

Iron overload, ROS accumulation, and perturbed metabolism are key factors in the initiation of ferroptosis. Sirt4 is a mitochondrial sirtuin that is highly expressed in the liver [68] and is involved in DNA damage repair and cellular metabolism [69]. Sirt4 blocks glutamine catabolism [70], which is required for the execution of ferroptosis [71, 72]. Sirt4 is also considered to be the key regulator in the antioxidative defense system, and depletion of Sirt4 can induce the accumulation of ROS by decreasing the activity of antioxidant enzymes in bovine mammary epithelial cells [73]. These findings indicate the potential correlation between ferroptosis and Sirt4. In this work, we identified Sirt4 as an important downstream effector of AGER1 in ferroptosis. In line with the abovementioned reports, Sirt4 deficiency severely impaired the antioxidant capacity, causing the accumulation of ROS and lipid peroxidation, eventually leading to ferroptosis. The depletion of Sirt4 also causes disturbances in iron metabolism and increases intracellular free iron by regulating the expression of the iron-dependent proteins TFR-1 and FTH, which triggers the onset of ferroptosis. Sirtuins are a family of NAD(+)-dependent protein deacetylases responsible for regulating multicellular and physiological functions [74]. The activity of sirtuins is associated with the availability of the coenzyme NAD(+) [75]. AGER1 has been verified to promote the intracellular production of NAD(+) [27, 76]. We found that the expression of Sirt4 was decreased by the knockdown of AGER1 and was increased with AGER1 overexpression, indicating that Sirt4 is likely the downstream target gene of AGER1. The co-IP assay further demonstrated the potential interaction between AGER1 and Sirt4. Additionally, the simultaneous knockdown of Sirt4 essentially abolishes the inhibitory effects of ferroptosis observed in AGER1-overexpressing cells, further indicating that the inhibitory role of AGER1 in regulating ferroptosis is dependent on Sirt4. Given the above, we speculate that the inhibitory effect of AGER1 on ferroptosis is dependent on the regulation of Sitr4.

In conclusion, the present work showed that ferroptosis contributes to the pathogenesis of liver fibrosis in NASH with T2DM by promoting hepatocyte EMT, and that AGER1 can ameliorate liver fibrosis through inhibiting ferroptosis by regulating Sirt4. Therefore, targeting AGER1 will be a promising strategy for the treatment of liver fibrosis in patients with NASH combined with T2DM.

## Experimental models and subject details

### Animal

Male C57BL/6L mice of 5 weeks of age (20–22 g) were purchased from Tianjin Medical University Laboratory Animal Center. All mice were housed in specific pathogen-free conditions with a 12 h light/dark cycle at 22–25 °C and free access to food and water. Prior to the experimentation, all animals were accustomed to the environment for 1 week. The body weight and blood glucose levels were monitored every week. Mice were starved for 8 h before being sacrificed and the livers were rapidly removed, weighed, and preserved in liquid nitrogen for subsequent analysis. All animal experiment protocols were approved by the Animal Care and Use Committee of Tianjin Medical University Chu Hsien-I Memorial Hospital.

### HFD-induced mice model

The high-fat diet (HFD) model was constructed based on the feeding of mice on an HFD diet (HFD: fat, 60% kcal; carbohydrate, 20% kcal; protein 20% kcal) (Sinodiets, Siping, China) for 19 weeks to induce NASH model. The additional mice were fed a standard diet of normal chow ((NC: carbohydrate, 70% kcal; protein, 20% kcal; fat, 10% kcal) (Medicience, Yangzhou, China) for 19 weeks to serve as a control group.

### HFD + DM mice model

After 12 weeks of HFD, mice were injected with a low dose of STZ to induce diabetes (HFD + DM). Mice were fasting for 12 h and then injected with STZ intraperitoneally (60 mg/kg dissolved in 0.1 M sodium citrate buffer (pH 4.5), SigmaAldrich, St. Louis, MO, USA) for 2 consecutive days, while a standard volume of citrate buffer was administered to the NC group. The levels of blood glucose were measured for one week following STZ injections, mice with random blood glucose concentrations >16.7 mmol/L (300 mg/dL), accompanied by polydipsia, polyuria, and polyphagia, were considered diabetic and then fed with HFD for 6 weeks additionally.

### Ferrostatin-1 treatment

To determine the protective effects of Fer-1 on HFD + DM model-induced liver fibrosis, after the HFD + DM model established, mice were received intraperitoneal injections of Fer-1 (1 mg/kg/d, dissolved in 2% DMSO, Selleck, Houston, TX, USA) every day, followed by feeding with HFD for 6 weeks additionally.

### Mouse adeno-associated virus9 injection

To overexpress AGER1 in vivo experiments, after the HFD + DM model established, mice were injected with adeno-associated virus serotype 9 (AAV9)-AGER1 (referred to as AAV-AGER1) by tail vein with 200 μL of virus containing 2 × 10^11^ vg of vectors, the empty vector (AAV-Control) was injected into mice as a negative control group and then fed with HFD for additional 6 weeks. The AAVs used above were packaged and purified by Genechem Co., LTD. (Shanghai, China).

### Cell culture and treatment

LO2 cells were purchased from ATCC (Manassas, VA, USA), and through mycoplasma detection and human cell line authentication by STR analysis. LO2 cells were maintained in Dulbecco’s Modified Eagle Medium (DMEM; Gibco, Carlsbad, CA, USA) complemented with 10% fetal bovine serum (FBS; Biological Industries, Cromwell, CT, USA) and 1% penicillin–streptomycin (Solarbio, Beijing, China) and all were cultured in an incubator at 37 °C with 5% CO_2_. In order to construct a cell model of lipid accumulation, LO2 cells were incubated with medium containing 110 μM palmitic acid (PA; Sigma-Aldrich, Saint Louis, MO, USA) for 24 h. To determine the effects of high glucose on steatotic hepatocytes, LO2 cells were cultured with 110 μM PA for 24 h, followed by 33.3 mM glucose for 24 h. To exclude the effect of osmolality, LO2 cells were cultured in DMEM with 5.5 mmol/L glucose, 24.5 mmol/L mannitol, 10% FBS, and 1% penicillin-streptomycin. For the Fer-1 group, LO2 cells were cultured with 110 μM PA for 24 h, followed by 33.3 mM glucose combined with 1 μM Fer-1 (Selleck, Houston, TX, USA) for 24 h. LO2 cells were cultured with DMSO of the same solubility to exclude the influence of the solvent. For the RSL3 group, LO2 cells were cultured with 110 μM PA for 24 h, followed by 0.1 μM RSL3 (APExBIO, Houston, TX, USA) for 24 h. LO2 cells were cultured with DMSO of the same solubility to exclude the influence of the solvent.

### Small interfering RNA (siRNA) or plasmid transfection

SiRNA specifically targeting AGER1 and Sirt4 were synthesized and designed by Genepharma (Shanghai, China). LO2 cells were cultured in 24-well plates, in the presence of 50–60% confluence, 50 pmol of each AGER1 siRNA or control siRNA was transfected into cells by using TransIntro® EL (TransGen Biotech, Beijing, China). Following 6 h of transfection, the medium was removed and replaced with 110 mM PA cultured for 24 h, followed by 33.3 mM glucose combined with or without 1 μM Fer-1 for 24 h.

AGER1 expression construct and vector were purchased from Genechem. LO2 cells were cultured in 24-well plates, in the presence of 70% confluence, 0.8 µg of each AGER1 expression conduct or vector was transfected into cells by using TransIntro® EL. Following 6 h of transfection, the medium was removed and replaced with 110 mM PA cultured for 24 h, followed by 33.3 mM glucose for 24 h. For the cotransfection study, the AGER1 expression construct or the vector was cotransfected with Sirt4 siRNA or control siRNA into LO2 cells by using TransIntro® EL. Following 6 h of transfection, the medium was removed and replaced with 110 mM PA cultured for 24 h, followed by 33.3 mM glucose for the other 24 h.

### Method details

#### Histological staining and immunohistochemistry

To conduct histologic and immunohistochemical analysis, liver tissues were fixed in 4% paraformaldehyde, embedded in paraffin, and then sectioned at a thickness of 5 μm. The liver sections were stained with hematoxylin and eosin (H&E; Solarbio, Beijing, China) to evaluate the ballooning degeneration and lobular inflammation. Sirius Red staining (Solarbio, Beijing, China) was performed to determine liver collagen content in accordance with the manufacturer’s instructions. To determine liver lipid accumulation, sections of OCT-embedded frozen tissues were stained with Oil red O stain (Solarbio, Beijing, China) under the manufacturer’s instructions. For the immunohistochemistry experiments, embedded sections were deparaffinized and rehydrated before incubated with primary antibodies including anti-α-SMA (A17910, ABclonal, dilution 1:200), anti-Vimentin (A2584, ABclonal, dilution 1:200), anti-E-cadherin (Cat.#: AF0131, Affinity, dilution 1:200), anti-GPX4 (Cat.#: DF6701, Affinity, dilution 1:200), anti-SLC7A11 (Cat.#: DF12509, Affinity, dilution 1:200), anti-FTH (Cat.#: DF6278, Affinity, dilution 1:200), anti-TFR-1 (Cat.#: AF5343, Affinity, dilution 1:200), anti-AGER1 (A9056, ABclonal, dilution 1:200) at 4°C overnight, and then incubated with anti-rabbit secondary antibody Goat Anti-Rabbit IgG (H + L) HRP (Cat.#: S0001, Affinity, dilution 1:200) or anti-mouse secondary antibody Goat Anti-Mouse IgG (H + L) HRP (Cat.#: S0002, Affinity, dilution 1:200). The sections were evaluated under a light microscope and the staining was quantified using Image-Pro Plus 6.0.

### Mouse lipid metabolic and liver function assays

Blood samples were collected from anesthetized mice’s posterior venous plexus, the serum was separated by centrifugation for 15 min at 3500 *g* and then stored at −80 °C for further analysis. The level of serum total TG, ALT, and AST were measured by using an Automatic biochemical analyzer in the clinical laboratory of Chu Hsien-I Memorial Hospital of Tianjin Medical University.

### Determination of intra-hepatic or intracellular iron, glutathione (GSH), and lipid peroxidation

Intra-hepatic and intracellular iron, GSH, and lipid peroxidation concentrations were detected by Ferrous Ion Content Assay Kit (Solarbio, Beijing, China), GSH Assay Kit (Solarbio, Beijing, China), and MDA Assay Kit (Solarbio, Beijing, China) on snap-frozen liver tissue or the collected cells following the manufacturer’s instructions.

### Protein isolation and western blot analysis

Liver tissues or cells were homogenized in RIPA lysis buffer with a cocktail of 1% protease (Solarbio, Beijing, China) and 1% phosphatase inhibitors (Solarbio, Beijing, China). Supernatants were collected by centrifugation at 15,000 *g* and 4 °C for 10 min. The concentration of protein was measured with an Instant BCA protein assay kit (EpiZyme, Shanghai, China). The protein of cells or tissues were separated by 7.5%, 10%, or 12% sodium dodecyl sulfate-polyacrylamide gel electrophoresis (SDS-PAGE) system in equal amounts and then transferred to an NC membrane (Merck, USA). TBST containing 5% skim milk was used to block membranes for 2 h, followed by overnight incubation with primary antibodies at 4 °C, and then incubated with secondary antibodies for 1 h at RT. Immunoblotting bands were captured using Tanon 5200 luminescent imaging system instrument (Tanon, Shanghai, China). Protein expressions were quantified by Imagine J and represented as a fold of the control. Antibodies were as follows: anti-α-SMA (A17910, ABclonal, dilution 1:1000), anti-Vimentin (A2584, ABclonal, dilution 1:1000), anti-E-cadherin (Cat.#: AF0131, Affinity, dilution 1:1000), anti-GPX4 (Cat.#: DF6701, Affinity, dilution 1:1000), anti-SLC7A11 (Cat.#: DF12509, Affinity, dilution 1:1000), anti-FTH (Cat.#: DF6278, Affinity, dilution 1:1000), anti-TFR-1 (Cat.#: AF5343, Affinity, dilution 1:1000), anti-AGER1 (A9056, ABclonal, dilution 1:1000), anti-Sirt4 (66543-1-Ig, Proteintech, dilution 1:1000), Goat Anti-Rabbit IgG (H + L) HRP (Cat.#: S0001, Affinity, dilution 1:1000), Goat Anti-Mouse IgG (H + L) HRP (Cat.#: S0002, Affinity, dilution 1:1000).

### RNA extraction and quantitative real-time polymerase chain reaction (qPCR)

The total RNA of liver tissues or LO2 cells was isolated and purified by using RNA Tissue/Cell Rapid Extraction Kit (Sparkjade Science Co., Ltd., China) in accordance with the manufacturer’s instruction. RNA quality was determined by the 260/280 absorbance radio on a Micro-spectrophotometer nano-300 (Allsheng, Hangzhou, China). The mRNA was reverse-transcribed into complementary DNA by using the HiFiScript cDNA Synthesis Kit (CoWin Biosciences, Taizhou, China) in a volume of 20 μL. qPCR was conducted by using SYBR Green (ABclonal, Wuhan, China, RK21203) in a QuantStudio 3 Flex Real-Time PCR System (Thermo Fisher). The target gene expression was quantified via the 2^−ΔΔCT^ method, and GAPDH was used as the endogenous control. The specific primers used in this experiment were provided in the Supplementary Table.

### Cell viability assay

Cell viability was performed with a Cell Counting Kit-8 (CCK-8) assay kit (Topscience, Shanghai, China). After seeding into 96-well plates, LO2 cells were incubated with the corresponding treatments. Afterward, each well was added with 10 μL CCK8 solutions and incubated for 2 h in an incubator at 37 °C with 5% CO_2_. Cell viability was calculated by the absorbance of 450 nm by using a microplate reader (Synergy HT, Bio-Tek, United States).

### Determination of intracellular liver enzyme and triglyceride

Intracellular AST, ALT, and TG concentrations were detected by Micro Glutamic-oxalacetic Transaminase Assay Kit (Solarbio, Beijing, China), Micro Glutamic-pyruvic Transaminase Assay Kit (Solarbio, Beijing, China), and Triglyceride Assay Kit (Nanjing Jiancheng Bioengineering Institute, China) on the collected cells following the manufacturer’s instructions.

### Determination of intracellular reactive oxygen species (ROS)

Intracellular ROS were detected by 2′,7′-dichlorofluorescein diacetate (DCFH-DA; Nanjing Jiancheng Bioengineering Institute, China) in accordance with manufacturer’s instructions. In brief, LO2 cells were seeded in 24-well plates, incubated with the corresponding treatments, afterwards 10 μM of DCFH-DA was added to each plate, incubating for 40 min at 37 °C in the dark. Fluorescence intensity was detected by a fluorescence microscope (Olympus, Tokyo, Japan).

### Immunofluorescence (IF) staining assays

LO2 cells were incubated with corresponding treatments, afterwards fixed with 4% paraformaldehyde for 15 min, followed by 0.2% Triton for 10 min to permeabilize the cell membrane. 1% Bovine Serum Albumin (BSA, solarbio, China) was used to block for 30 min, followed by overnight incubation with primary antibodies at 4 °C. On the next day, cells were incubated with CY3 goat anti-rat IgG (H + L) secondary antibody (GB21303, Servicebio, Wuhan, China) for 1 h in the dark. 4,6-diamidino-2-phenylindole (DAPI; Solarbio, Beijing, China) was used to stain the cell nucleus. Fluorescence intensity was visualized by a fluorescence microscope (Olympus, Tokyo, Japan).

### Mitochondrial morphological analysis

The collected LO2 cells were fixed with 2.5% glutaraldehyde (Alfa Aesar, Ward Hill, MA, USA) in 0.1 M phosphate buffer (pH 7.4) for 3 h at 4 °C. Afterward, 2% osmium tetroxide and 0.1 M sodium cacodylate (pH 7.4) were incubated with the cells for 2 h. Cells were dehydrated in acetone, embedded in Embed 812 resin, and ultrathin sections of the embedded resin were cut and stained with uranyl acetate and lead citrate. The ultrastructure of mitochondria was viewed by transmission electron microscopy (HT7700-SS; HITACHI, Tokyo, Japan).

### Coimmunoprecipitation (co-IP) analysis

The interaction between AGER1 and Sirt4 was determined by a co-IP assay. In brief, 50 μl Protein A/G Magnetic Beads (MedChemExpress, Monmouth Junction, NJ, USA) were incubated with the antibody against AGER1 or rabbit IgG for 1.5 h at RT. The LO2 cells were collected and lysed with RIPA buffer supplemented with PMSF and protease inhibitor cocktail. The cell lysates were incubated with the Protein A/G Magnetic Beads which were bound to the antibody against AGER1 or rabbit IgG, followed by rotation overnight at 4 °C. The immunocomplex was collected and then subjected to SDS-PAGE and immunoblotting.

### Quantification and statistical analysis

Data were represented as mean ± SD. All data were analyzed by Prism 8.0 (GraphPad, San Diego, CA). Comparisons between two groups were conducted using Unpaired Student’s t-tests. Comparisons among multiple groups were conducted using one-way (for one independent variable) analysis of variance. *P* < 0.05 was considered to be statistically significant.

## Data accessibility

The data that support the findings of this study are available from the corresponding author upon reasonable request.

## Supplementary information

Supplementary Information

Original western blots

Reproducibility checklist
