# Supplementary material for: AGER1 deficiency-triggered ferroptosis drives fibrosis progression in nonalcoholic steatohepatitis with type 2 diabetes mellitus
Source: Cell Death Discov. 2023 Jun 6;9:178. doi: 10.1038/s41420-023-01477-z (PMC10244405; doi:10.1038/s41420-023-01477-z)
Supplement: Supplementary file 1 — Supplementary Information [file 41420_2023_1477_MOESM1_ESM.docx]

**Supplementary material**

**Table. S1 Summary of primer sequences used for RT-PCR**

| **Gene (Human) Forward Primer (5' - 3 ') Reverse Primer (5' - 3')** |
| --- |
| AGER1 CGACTTCTTCAGCGACTCCTTCTTC TGCCTGTCTGGGAATACCTCTGG  Sirt4 TATCCTCACTGCCTGGGAGAAGAAG AGTTTCAGACACGCCAAGTCATCC  E-cadherin GATTCTGCTGCTCTTGCTGTTTCTTC GGTCCTCTTCTCCGCCTCCTTC  Vimentin TCGTGAATACCAAGACCTGCTCAATG AATCCTGCTCTCCTCGCCTTCC  α-SMA GCGTGGCTATTCCTTCGTTAC CATAGTGGTGCCCCCTGATAG  GPX4 GGCTTCGTGTGCATCGTCACC TTCACCACGCAGCCGTTCTTG  SLC7A11 CGCAAGCACACTCCTCTACCAG TCAGAGTGATGACGAAGCCAATCC  TFR-1 TGTGGCGTATAGTAAGGCTGCAAC GGCAATCCTGATGACCGAGATGG  FTH AGAACTACCACCAGGACTCAGAGG GGAAGATTCGGCCACCTCGTTG  GAPDH GGCACCGTCAAGGCTGAGAAC GGTGGCAGTGATGGCATGGAC  Si-AGER1 GACCAUCACAACUAUGACATT UGUCAUAGUUGUGAUGGUCTT  Si-NC UUCUCCGAACGUGUCACGUTT ACGUGACACGUUCGGAGAATT  Si-Sirt4 CAUCCAGCAUGGUGAUUUUTT AAAAUCACCAUGCUGGAUGTT  Si-NC UUCUCCGAACGUGUCACGUTT ACGUGACACGUUCGGAGAATT |
| **Gene (Mouse) Forward Primer (5' - 3 ') Reverse Primer (5' - 3 ')** |
| AGER1 GCTGACACTGAGAACCTGCTGAAG GTCTGCCACCATTCCAACTCCTC  Sirt4 GCCTCAATTCTCCTCTCACCAACC CGTCCACGTTCTGAGTCACCAAC  E-cadherin ATCCTGACCAGCAGTTCGTTGTTG GTTCCTCGTTCTCCACTCTCACATG  Vimentin ACTAGCCGCAGCCTCTATTCCTC GAAGTCCACCGAGTCTTGAAGCAG  α-SMA CGTGGCTATTCCTTCGTGACTACTG CGTCAGGCAGTTCGTAGCTCTTC  GPX4 CATGCCCGATATGCTGAGTGTGG TAGCACGGCAGGTCCTTCTCTATC  SLC7A11 CCTCTGACGATGGTGATGCTCTTC GGTGCTGAATGGGTCCGAGTAAAG  TFR-1 GTTTCCGCCATCTCAGTCATCAGG GGACTTCGCCGCAACACCAG  FTH TGCCATCAACCGCCAGATCAAC ATTCAGCCCGCTCTCCCAGTC  IL-1β CACTACAGGCTCCGAGATGAACAAC TGTCGTTGCTTGGTTCTCCTTGTAC  IL-6 CTCCCAACAGACCTGTCTATAC CCATTGCACAACTCTTTTCTCA  TNF-α ATGTCTCAGCCTCTTCTCATTC GCTTGTCACTCGAATTTTGAGA  GAPDH AATGGTGAAGGTCGGTGTGAACG TCGCTCCTGGAAGATGGTGATGG |

**Supplementary Figures**

**
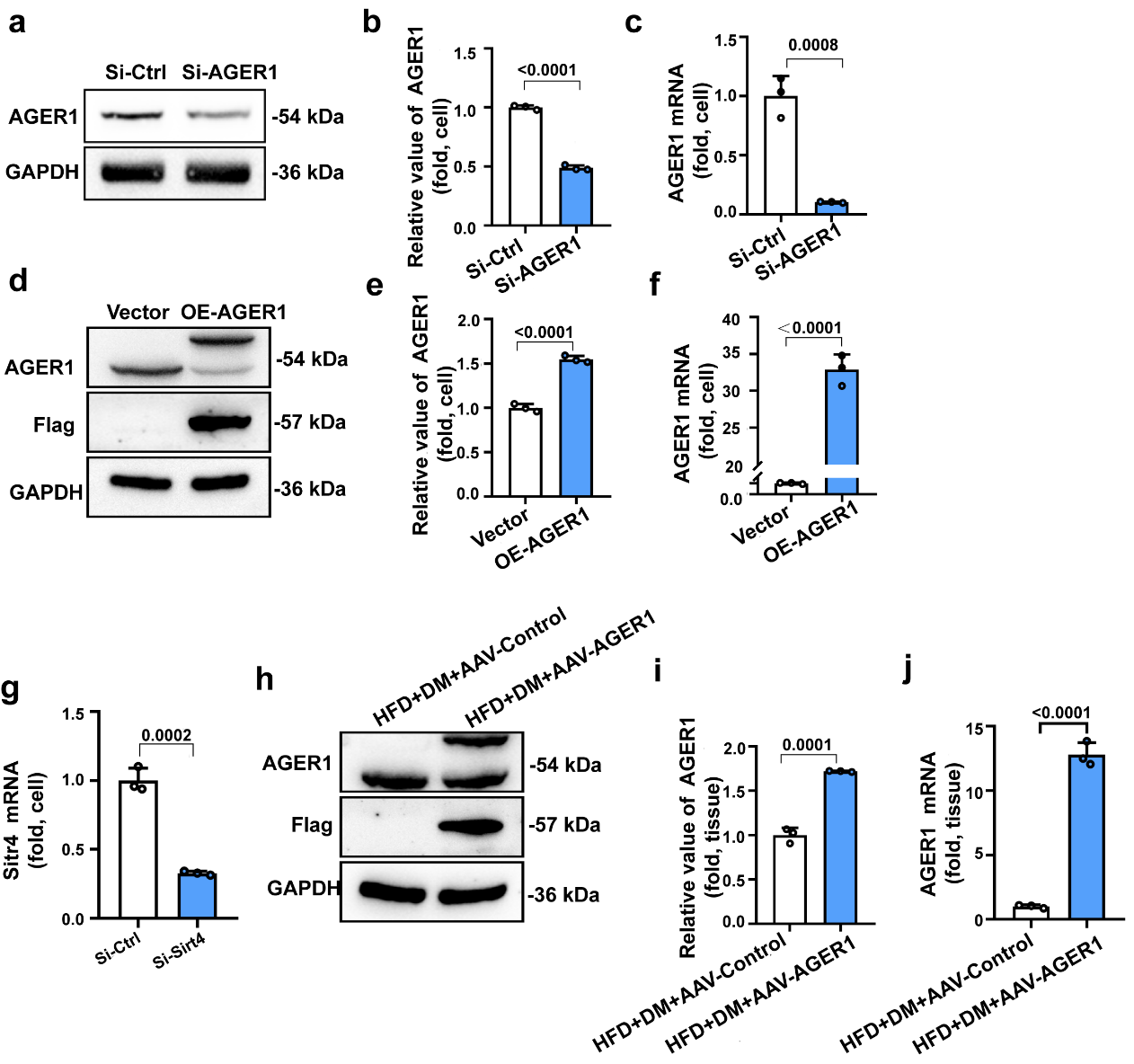
**

**Fig. S1 a,** **b** Representative immunoblotting bands and quantitative analysis for expression alterations of AGER1 in the cells of Si-Ctrl and Si-AGER1 groups. **c** The mRNA expression of AGER1 in cells of Si-Ctrl and Si-AGER1 groups. **d, e** Representative immunoblotting bands and quantitative analysis for expression alterations of AGER1 and Flag in the cells of Vector and OE-AGER1 groups. **f** The mRNA expression of AGER1 in the cells of Vector and OE-AGER1 groups. **g** The mRNA expression of Sirt4 in the cells of Si-Ctrl and Si-Sirt4 groups. **h, i** Representative immunoblotting bands and quantitative analysis for expression alterations of AGER1 and Flag in the livers of HFD+DM+AAV-Ctrl and HFD+DM+AAV-AGER1 groups. **j** The mRNA expression of AGER1 in the livers of HFD+DM+AAV-Ctrl and HFD+DM+AAV-AGER1 groups. Data are expressed as mean ± SD. All the above experiments were independently repeated at least three times. Statistical significance was tested by Student’s *t* test.
