# Supplementary material for: AGER1 deficiency-triggered ferroptosis drives fibrosis progression in nonalcoholic steatohepatitis with type 2 diabetes mellitus
Source: Cell Death Discov. 2023 Jun 6;9:178. doi: 10.1038/s41420-023-01477-z (PMC10244405; doi:10.1038/s41420-023-01477-z)

Fig.1

E-cadherin (120 kDa)

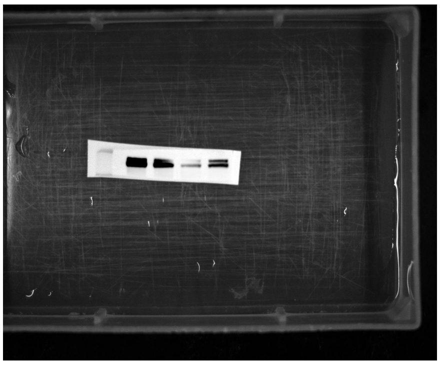

α-SMA (45 kDa)

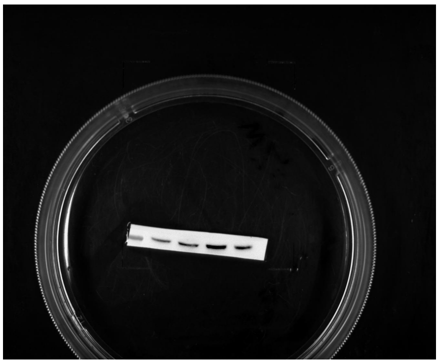

Vimentin (53 kDa)

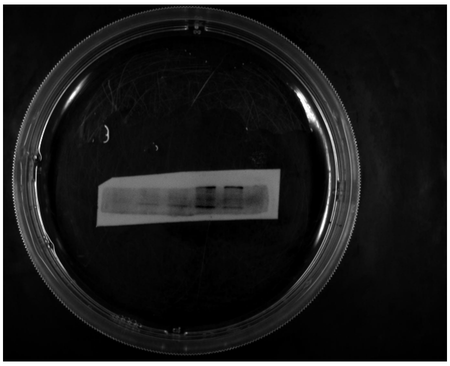

GAPDH (36 kDa)

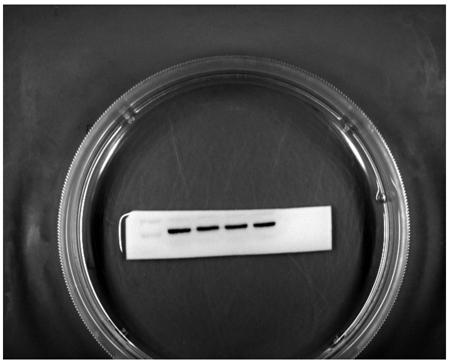

GPX4 (17 kDa)

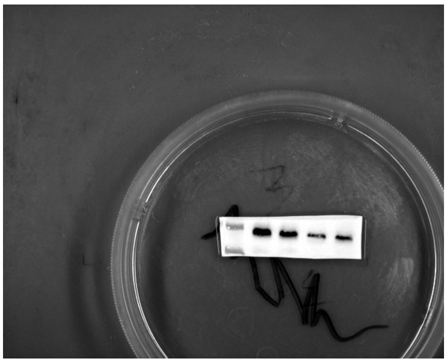

SLC7A11 (55 kDa)

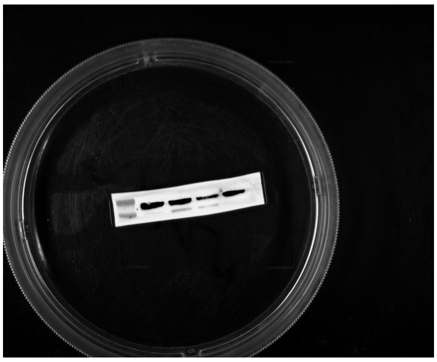

FTH (21 kDa)

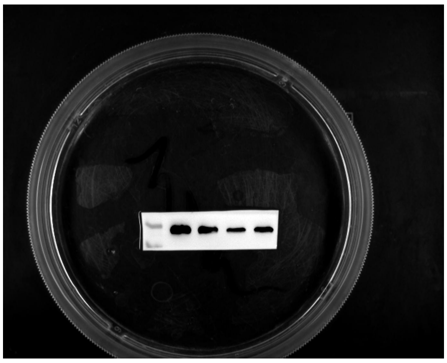

TFR-1 (100 kDa)

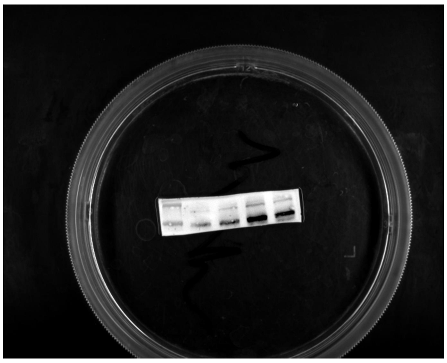

GAPDH (36 kDa)

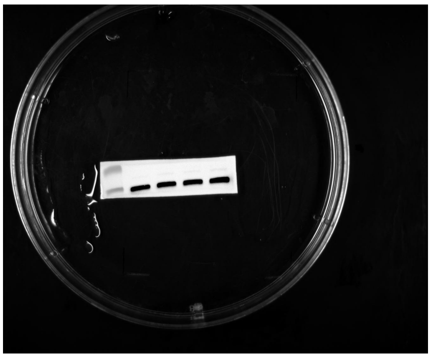

Fig.2

E-cadherin (120 kDa)

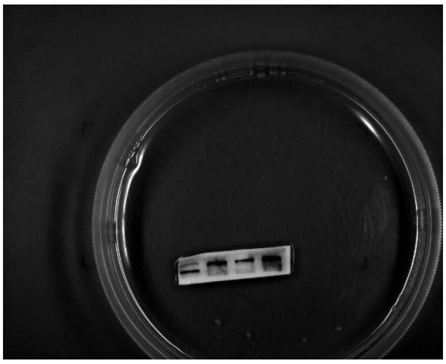

α-SMA (45kDa)

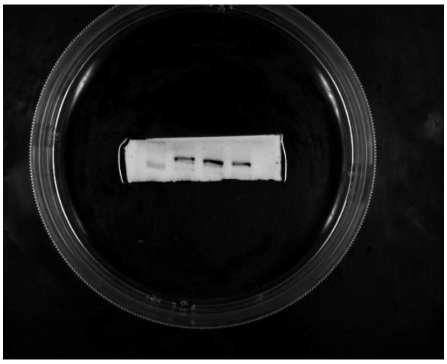

Vimentin (53 kDa)

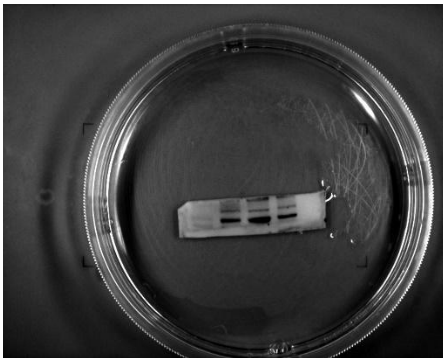

GAPDH (36 kDa)

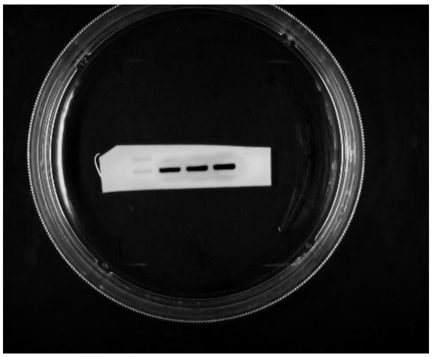

GPX4 (17 kDa)

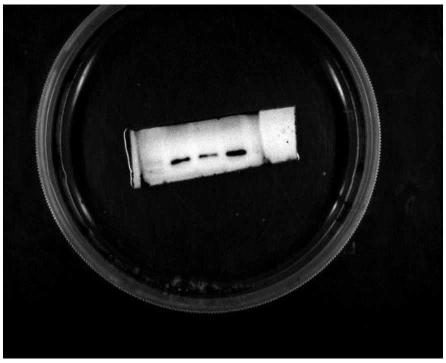

SLC7A11 (55 kDa)

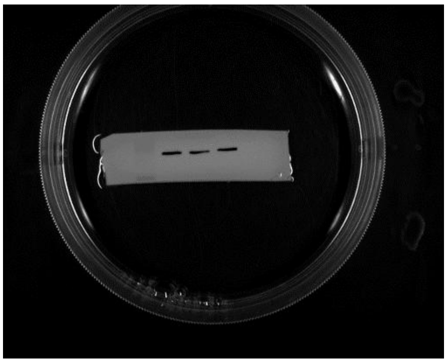

FTH (21 kDa)

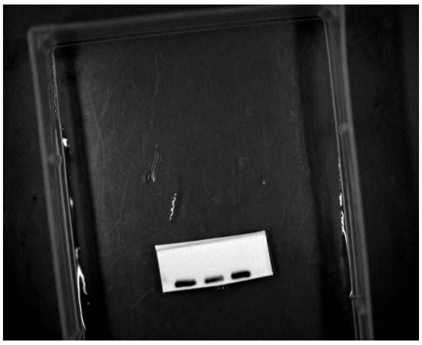

TFR-1 (100 kDa)

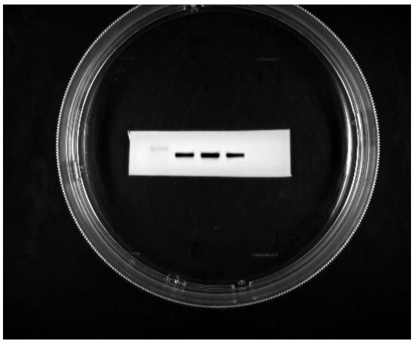

GAPDH (36 kDa)

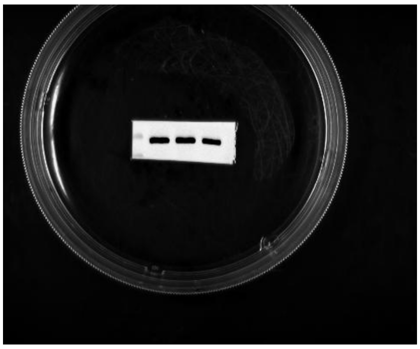

Fig.3

GPX4 (17 kDa)

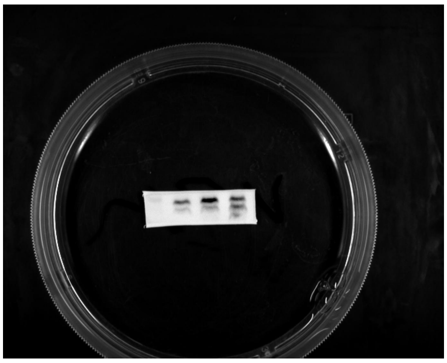

SLC7A11 (55 kDa)

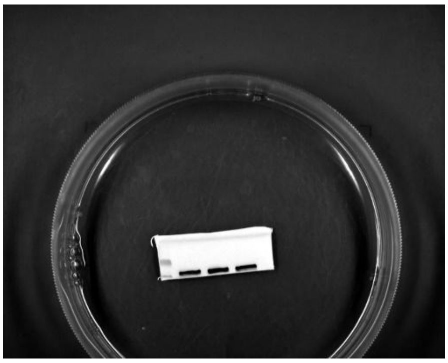

FTH (21 kDa)

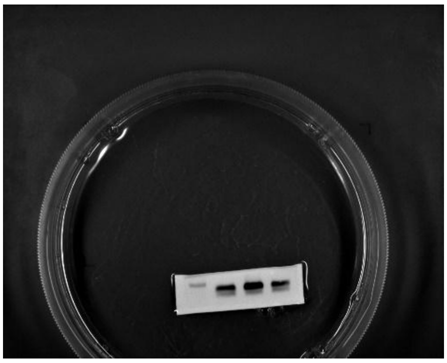

TFR-1 (100 kDa)

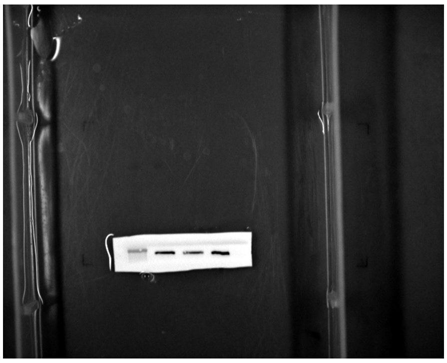

GAPDH (36 kDa)

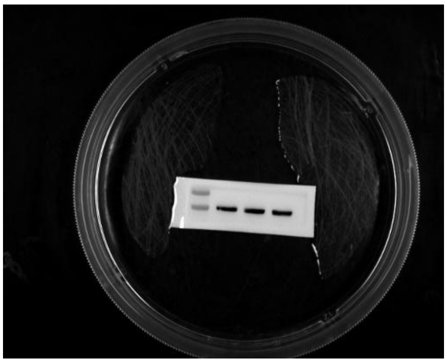

E-cadherin (120 kDa)

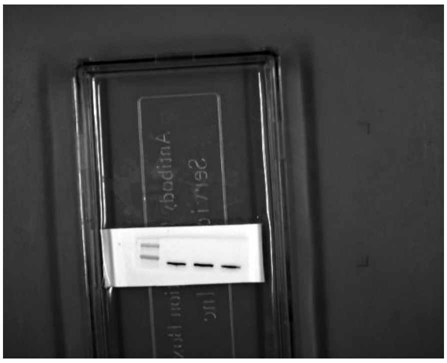

α-SMA (45 kDa)

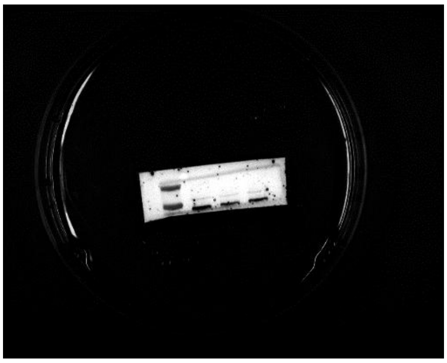

Vimentin (53 kDa)

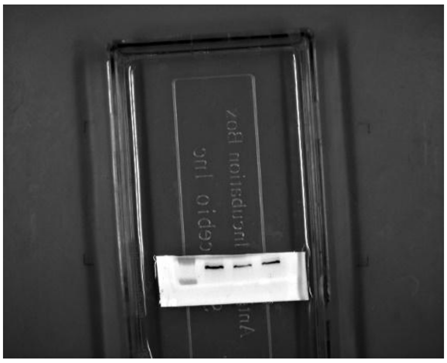

GPADH (36 kDa)

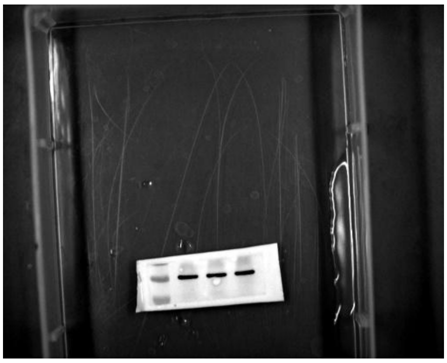

Fig.4

AGER1 (Fig.4c) (54 kDa)

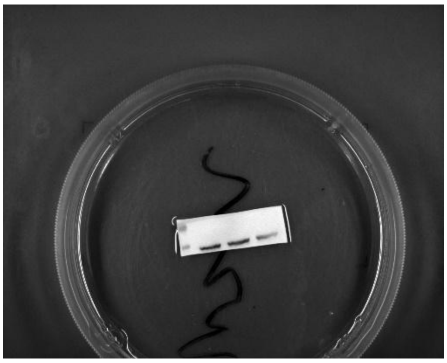

GAPDH (36 kDa)

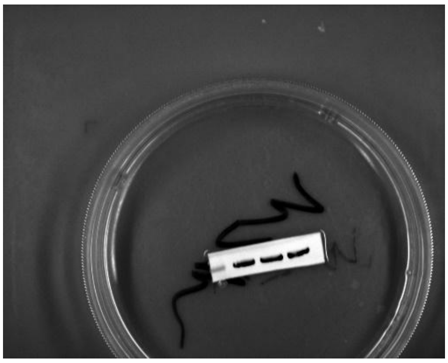

AGER1 (Fig.4e) (54 kDa)

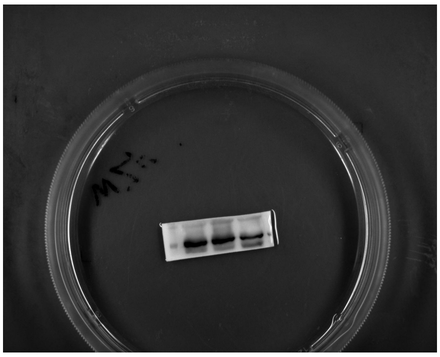

GAPDH (36 kDa)

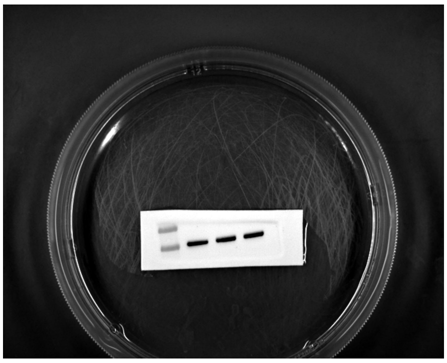

E-cadherin (120 kDa)

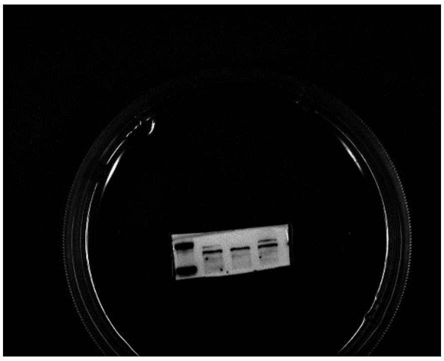

α-SMA (45 kDa)

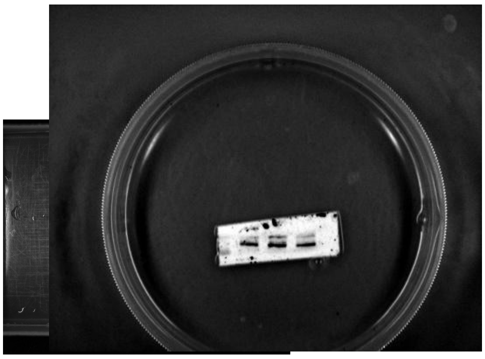

Vimentin (53 kDa) (the top band)

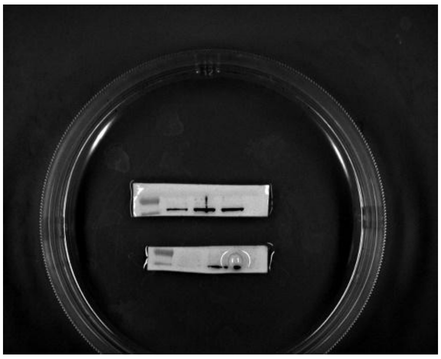

GAPDH (36 kDa)

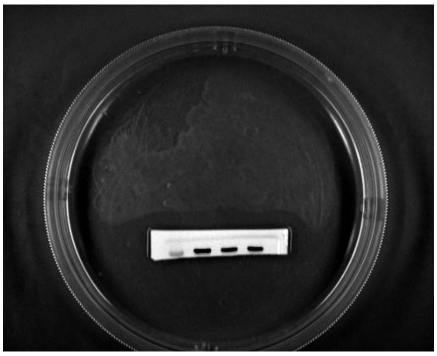

GPX4 (17 kDa)

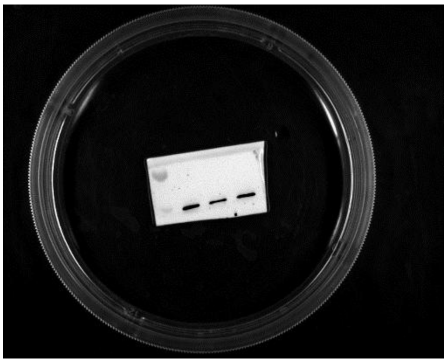

SLC7A11 (55 kDa)

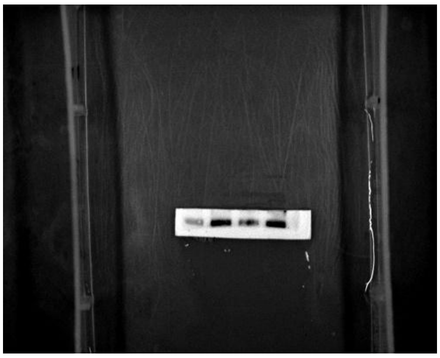

FTH (21 kDa)

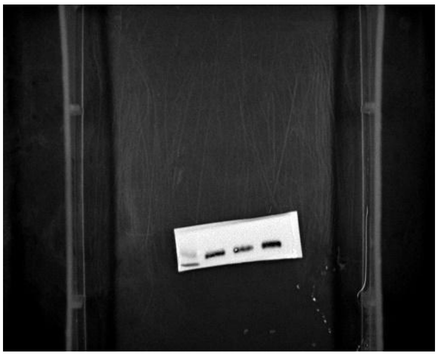

TFR-1 (100 kDa)

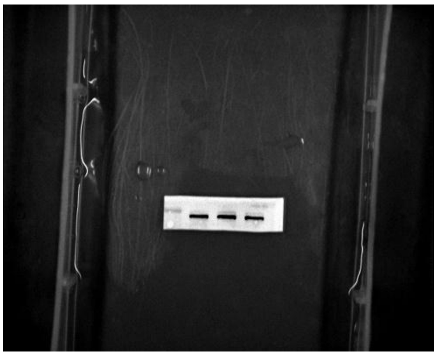

GAPDH (36kDa)

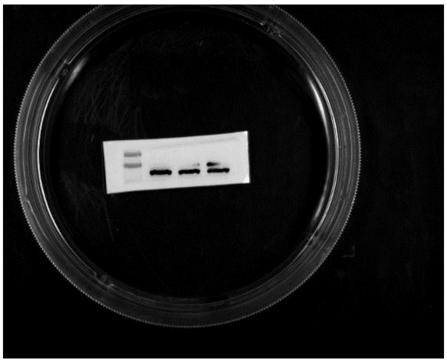

Fig.5

GPX4 (17 kDa)

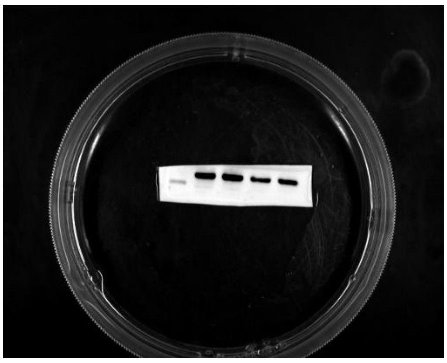

SLC7A11 (55 kDa)

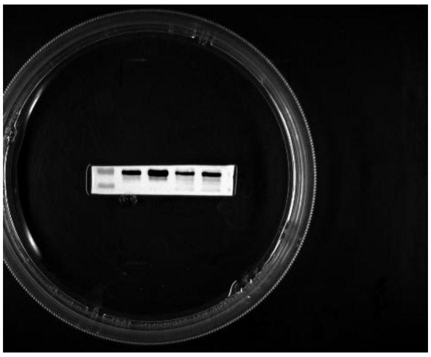

FTH (21 kDa)

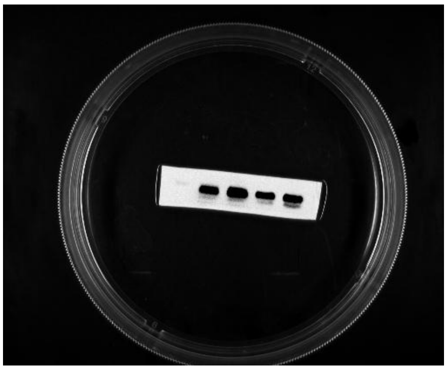

TFR-1 (100 kDa)

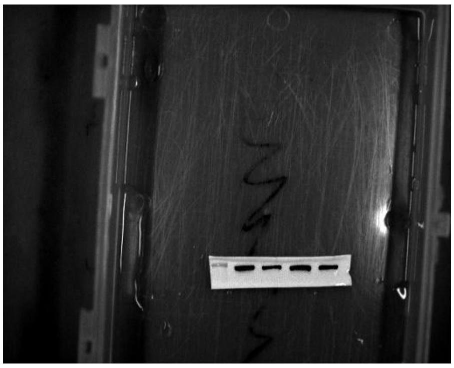

GAPDH (36 kDa)

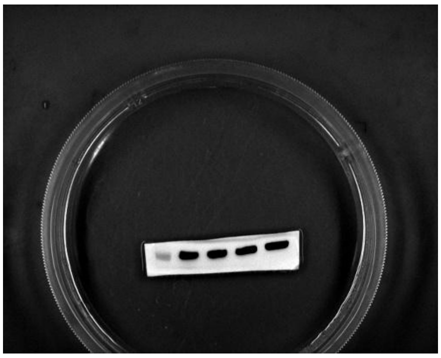

E-cadherin (120 kDa) (the bottom band)

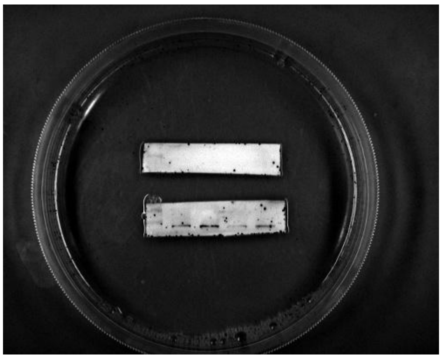

α-SMA (45 kDa)

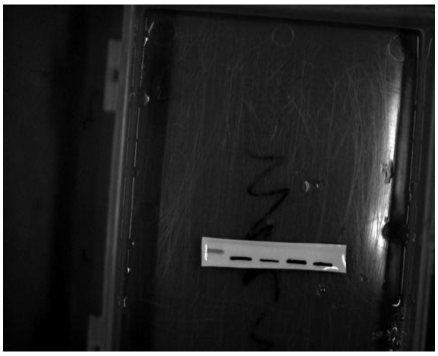

Vimentin (53 kDa)

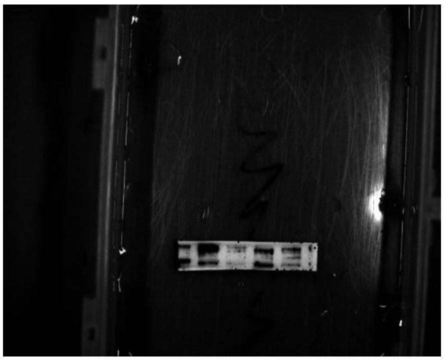

GAPDH (36 kDa)

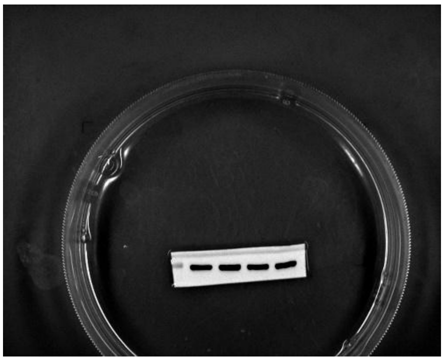

Sirt4 (Fig.5m) (35 kDa)

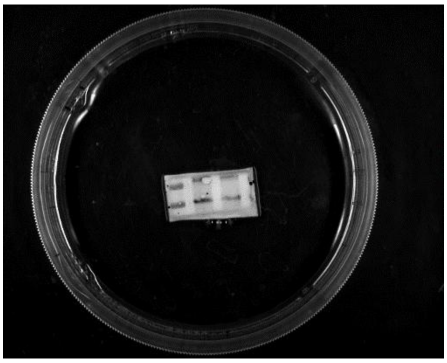

β-actin (42 kDa)

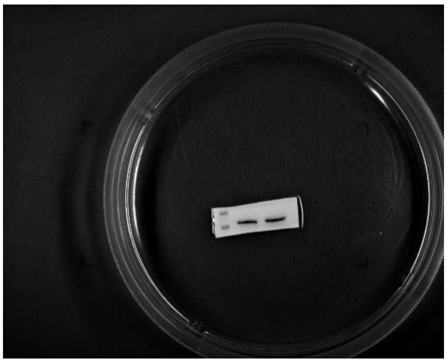

Sirt4 (Fig.5n) (35 kDa)

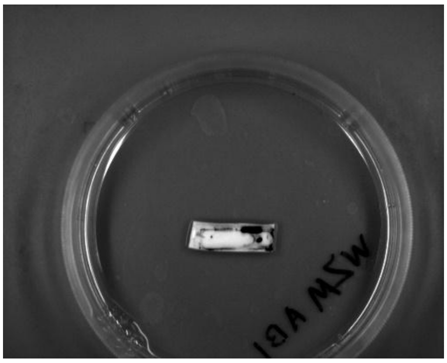

β-actin (42 kDa)

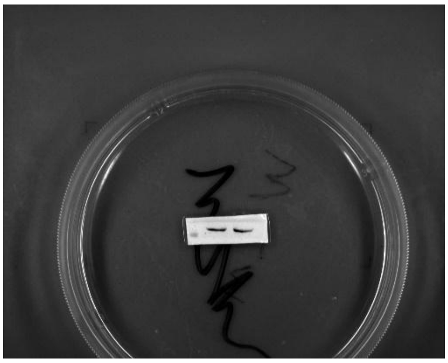

AGER1 (Fig.5o) (54 kDa)

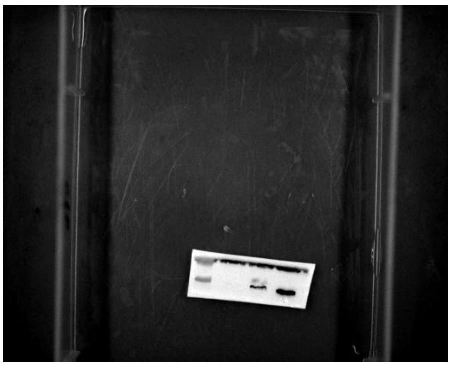

Sirt4 (Fig.5o) (35 kDa)

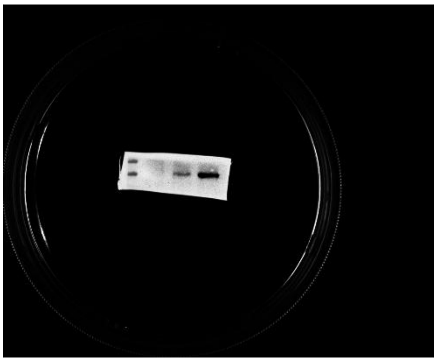

Fig.6

E-cadherin (120 kDa)

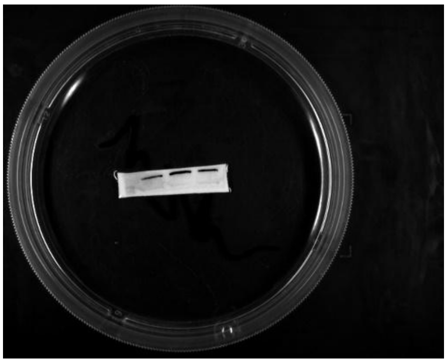

α-SMA (45 kDa)

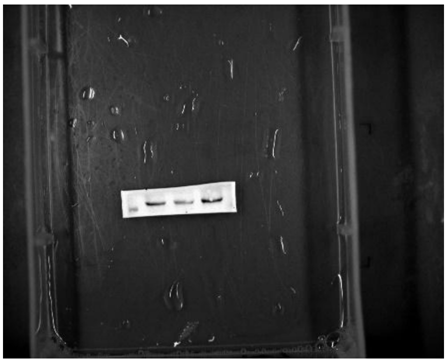

Vimentin (53 kDa)

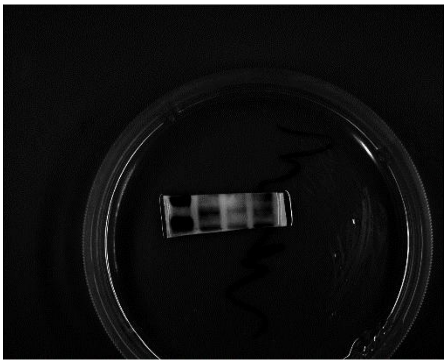

GAPDH (35 kDa)

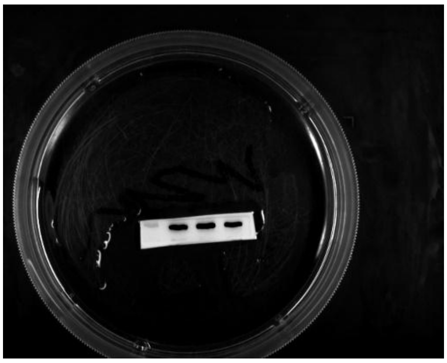

GPX4 (17 kDa)

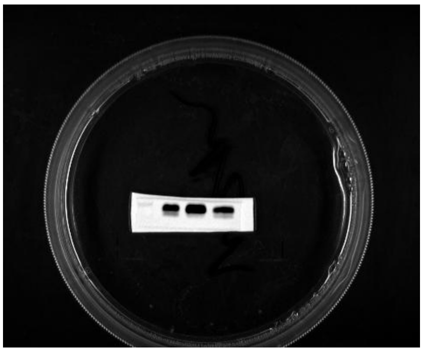

SLC7A11(55 kDa)

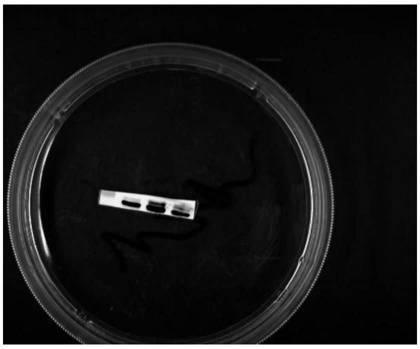

FTH (21 kDa)

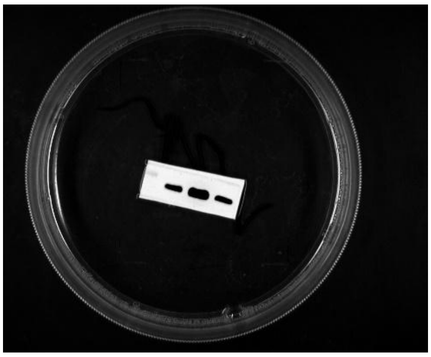

TFR-1 (100 kDa)

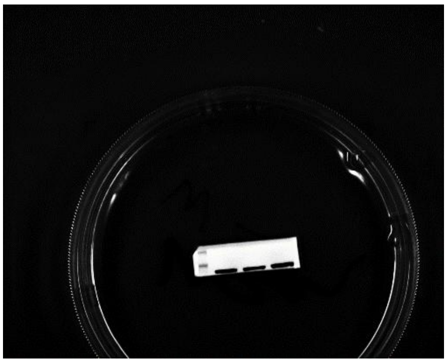

GAPDH (35 kDa)

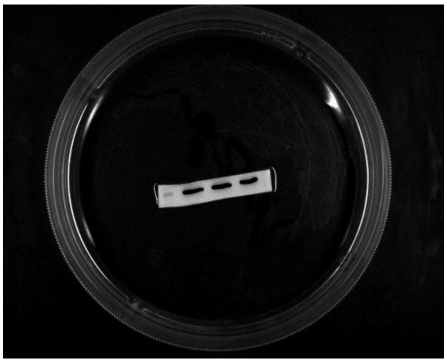

Supplementary Figure

AGER1 (Fig.1a) (54 kDa)

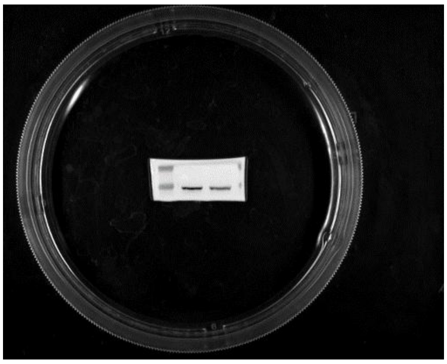

GAPDH (Fig.1a) (35 kDa)

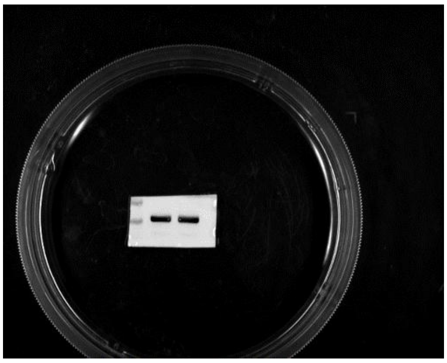

AGER1 (Fig.1d) (54 kDa)

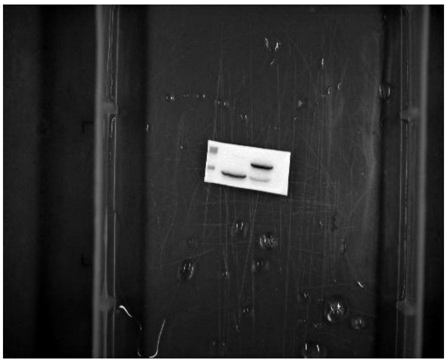

Flag (Fig.1d) (54 kDa)

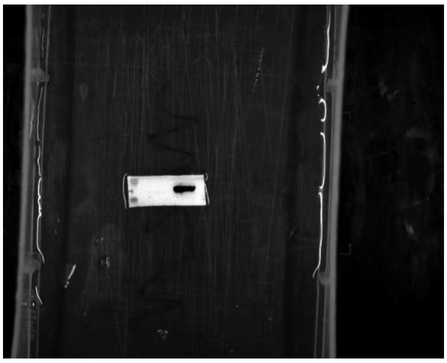

GAPDH (Fig.1d) (35 kDa)

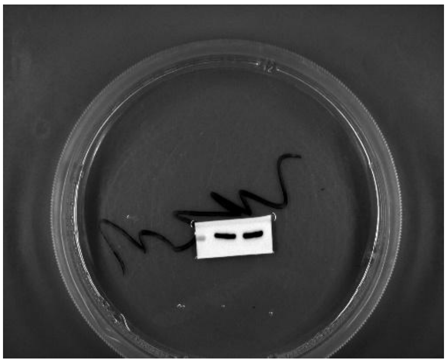

AGER1 (Fig.1h) (54 kDa)

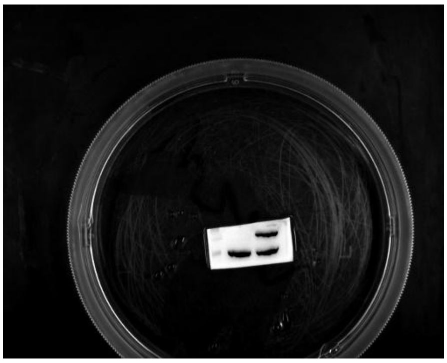

Flag (Fig.1h) (54 kDa)

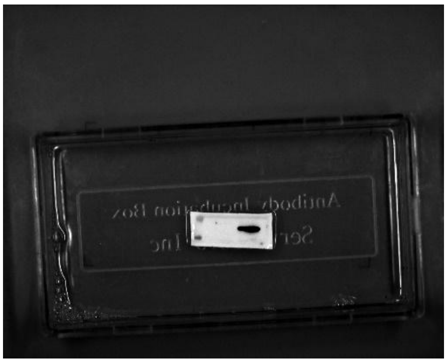

GAPDH (Fig.1h) (35 kDa)

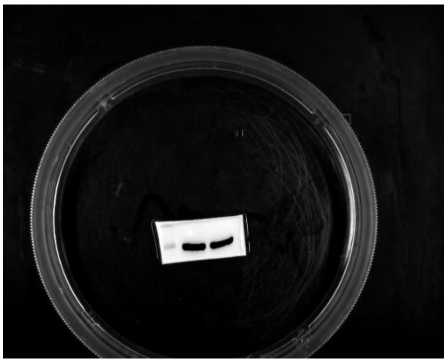

Supplement: Supplementary file 2 — Original western blots [file 41420_2023_1477_MOESM2_ESM.pdf]
